# Synthesis and biological evaluation of Combretastatin A-4 derivatives containing a 3’-O-substituted carbonic ether moiety as potential antitumor agents

**DOI:** 10.1186/1752-153X-7-179

**Published:** 2013-12-05

**Authors:** Mingyi Ma, Longru Sun, Hongxiang Lou, Mei Ji

**Affiliations:** 1 Department of Natural Products Chemistry, Key Lab of Chemical Biology (MOE), School of Pharmaceutical Sciences, Shandong University, No. 44 West Wenhua Road, Jinan 250012, PR China

**Keywords:** Combretastatin A-4, Synthesis, Antitumor activity, WST-1, Cell cycle arrest, Apoptosis

## Abstract

**Background:**

Combretastatin A-4 (CA-4), which is an excellent antineoplastic agent, was isolated from *Combretum caffrum*. To date, structural modification studies of CA-4 have focused predominantly on the construction of new therapeutic agents for drug discovery. As a part of our ongoing work towards the modification of natural products, we have focused on the 3’-O-substituent groups in the B-ring of CA-4 under the hypothesis that these novel derivatives will possess good bioactivities and behave as effective antiproliferative pro-drugs.

**Results:**

A series of novel CA-4 derivatives, which contained a 3’-O-substituted carbonic ether moiety, were synthesized and evaluated for their antitumor activities against four tumor cell lines, including MDA-MB-231, MCF-7, K562 and A549 cells. These derivatives exhibited clear antitumor activities, and CA-4E, in particular, showed the highest bioactivity of all of the derivatives tested against all four tumor cell lines, with IC_50_ values in the range of 1 to 180 nM. Based on its high bioactivity, CA-4E was subsequently selected to investigate the antitumor mechanism of these synthetic compounds. The cell cycle results demonstrated that CA-4E induced time- and dose-dependent G2/M arrest in a similar manner to CA-4, although its effect was more powerful than that of CA-4, and the apoptosis data showed that CA-4E induced cellular apoptosis in a dose-dependent manner.

**Conclusions:**

The newly synthesized CA-4 derivatives exhibited good antitumor activities *in vitro*, with CA-4E, in particular, showing the highest bioactivity of all of the compounds tested. Furthermore, CA-4E induced time- and dose-dependent G2/M arrest and cellular apoptosis in a dose-dependent manner. Taken together, these results suggest that CA-4E should be subjected to further investigation as a potential anticancer drug candidate.

## Background

**Figure 1:**
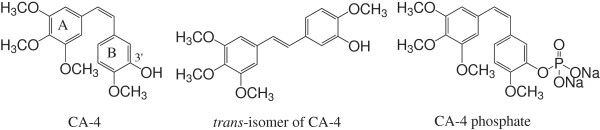
**Structures of CA-4, the *trans *** **isomer of CA-4 and CA-4 phosphate.**

Combretastatin A-4 (CA-4, Figure 1) is a natural product, which was first isolated from the bark of the South African tree *Combretum caffrum* by Pettit *et al*. in 1989 [1]. As a potentially effective vascular disrupting agent (VDA), CA-4 can discriminate between normal vessels and tumor vessels and selectively disrupt the abnormal tumor vasculature, leading ultimately to vascular collapse [2-4]. Furthermore, CA-4 exhibits excellent anticancer properties by interfering with the dynamics of tubulin, in that CA-4 inhibits the polymerization of tubulin by binding to the colchicine site, which results in cell mitotic arrest [5]. The activity of the *trans* isomer of CA-4 (Figure 1), however, is much lower than that of CA-4. For example, CA-4 displays cytotoxicity at low nanomolar concentrations, whereas the *trans* isomer inhibits cell growth in the micromolar range [6].

**Figure 2:**
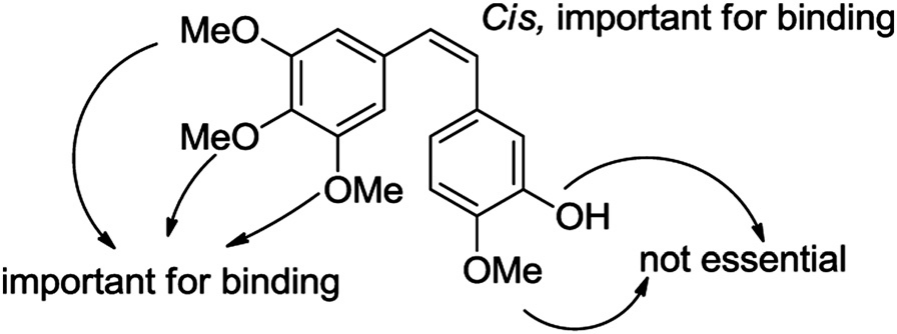
Structure-activity relationships of a tubulin binder.

CA-4 has recently been the subject of considerable interest from pharmaceutical chemists attempting to design new compounds capable of mimicking the bioactivity of CA-4, and a wide variety of analogues and derivatives of CA-4 have been synthesized and evaluated for their antitumor activities *in vitro*. Based on these derivatives, several structure–activity relationships (SARs) have been identified for CA-4, which are summarized in Figure 2. These SARs have demonstrated that the 3,4,5-trimethoxysubstituted A-ring and the *cis* configuration of the stilbene connecting the A-ring to the B-ring are both essential to the antiproliferative activity of these compounds [7-9]. The 3’-hydroxy group of B-ring has been shown to be non-essential for the interaction of CA-4 with tubulin, and this particular moiety could potentially be replaced with other suitable substituents [10]. A series of CA-4 derivatives bearing different substituents at the 3’-position of the B-ring, such as a halogen atom (e.g., fluorine or bromine), amine, boronic acid, nitro, amide, alkoxy or acyl group, were synthesized and evaluated for their antitumor activities [11-13]. Among these derivatives, a water soluble pro-drug of CA-4, CA-4 phosphate (CA-4P, Figure 1), was identified and progressed into clinical trials as a potential vascular disrupting agent that could be used in combination with conventional cytotoxic therapies for the treatment of cancer [14]. With this in mind, and as part of our ongoing work towards the synthesis of natural product derivatives with interesting biological properties, we have focused our attention on modifying the 3’-O-substituent groups in the B-ring of CA-4. It is envisaged that these novel derivatives would posses good bioactivities compared to CA-4, and that they could be used as potential antiproliferative pro-drugs. Herein, we describe the design, synthesis and evaluation of the cytotoxicity of a series of novel Combretastatin A-4 derivatives. Furthermore, the most active of these compounds, CA-4E, was selected to investigate the antitumor mechanism of this novel series of compounds.

## Results and discussion

### Chemistry

CA-4 is recognized as an important parent compound, in terms of its biological effects, and hundreds of structural modified CA-4 derivatives have been synthesized and reported [15,16], where the modifications have traditionally been affected using either a Wittig reaction or a Perkin condensation reaction. Using the Wittig reaction, the CA-4 product is generally formed as a mixture with the corresponding *trans* isomer, which can be difficult to remove, and this can lead to complications in any follow-up work [17]. In contrast, the Perkin condensation proceeds stereoselectively to afford CA-4 as the major product [18]. The use of the Perkin condensation reaction therefore provides better access to the CA-4 derivatives and, in contrast to the Wittig reaction, does not require extensive purification procedures.

**Scheme 1:**
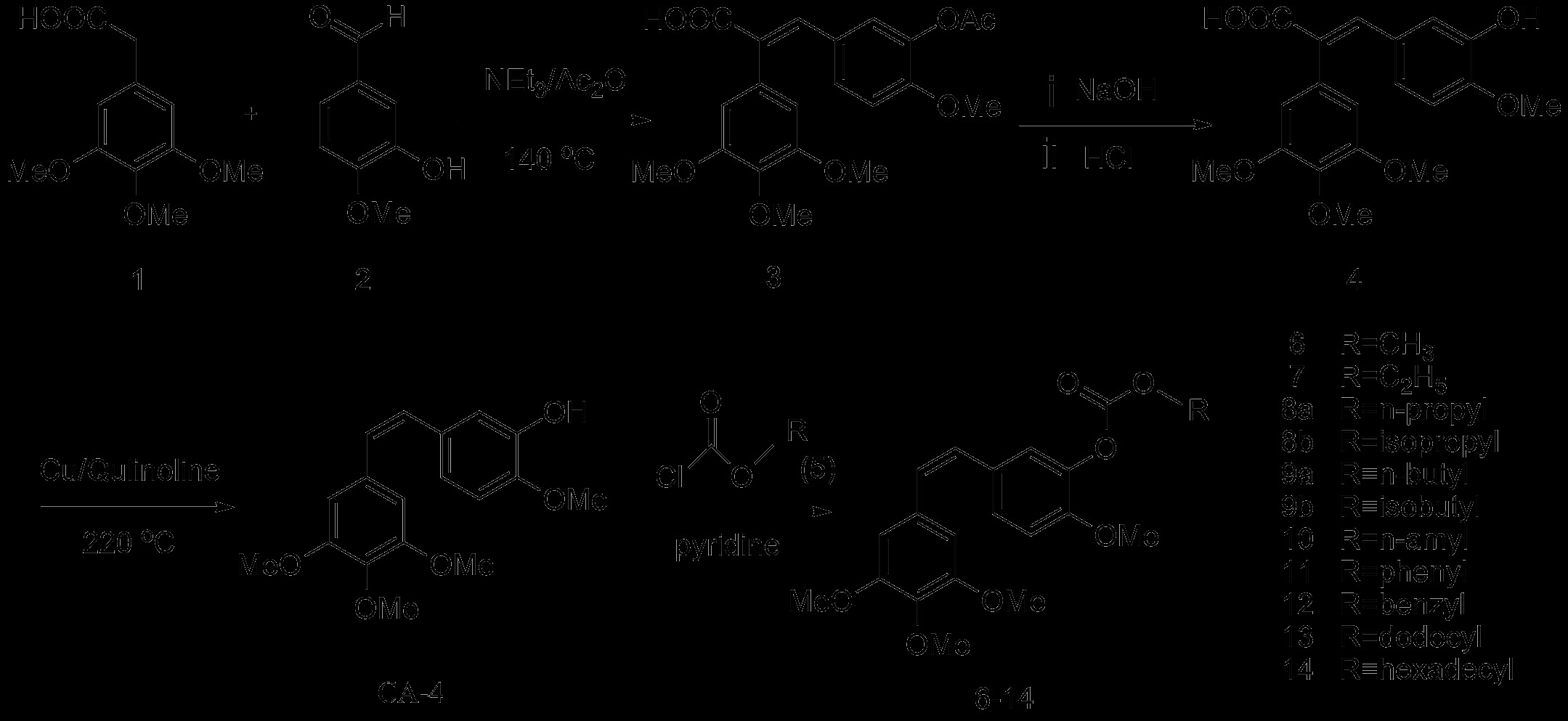
Synthetic route to CA-4 and the target compounds 6-14.

Our synthetic route to the CA-4 derivatives is depicted in Scheme 1 (Additional file 1), where the parent compound CA-4 was prepared via a Perkin condensation following a previously described procedure [18]. A mixture of 3,4,5-trimethoxyphenylacetic acid (**1**), 3-hydroxy-4-methoxybenzaldehyde (**2**), triethylamine, and acetic anhydride was heated at reflux at 140°C to give intermediate **3**. The reaction mixture was then hydrolyzed using sodium hydroxide, and the cinnamic acid analogue **4** was precipitated from the solution following acidification with concentrated hydrochloric acid. Subsequent purification of the crude precipitate by recrystallization from ethanol gave pure **4** in 65% yield. Compound **4** was then decarboxylated with copper powder in quinoline at 220°C, and the crude product was purified by flash column chromatography (FCC) to give CA-4 in 55% yield together with a very small amount of its *trans* isomer. The CA-4 derivatives **6**-**14**, which contained an O-substituted carbonic ether moiety, were prepared via the acylation of CA-4 with the chloroformic acid alkyl esters **5** and pyridine, and the crude derivatives were purified by FFC. All of the newly synthesized compounds were characterized using their spectra data (Additional file 2).

### Biological activity evaluation

#### WST-1-based cell cytotoxicity assay

The *in vitro* cytotoxicities of the synthesized compounds were evaluated against selected human cancer cell lines for breast (MDA-MB-231 and MCF-7), lung (A549) and leukemia (K562) using a WST-1-based colorimetric cell cytotoxicity assay [19]. All of the experiments were performed in triplicate. The WST-1-based assay was used in the current study instead of the MTT-based assay because it is generally considered to be more rapid, sensitive and accurate than the MTT assay. Furthermore, this assay involves the detection of water-soluble formazan, and therefore does not require an additional solubilization step [19]. The absorbance was measured on a microplate reader at 450/630 nm with CA-4 being used as a positive control. The corresponding IC_50_ values were calculated and are listed in Table 1.

**Table 1:** ***In vitro *** **cytotoxicities against four different tumor cell lines**

**Compound**	** IC**_**50 **_**(nM)**			
	**MDA-MB-231**	**MCF-7**	**K562**	**A549**
CA-4	6.27	10.49	5.75	428
**6**	12.29	8.00	7.68	693
**7**(CA-4E)	2.30	1.32	<2.50	180
**8a**	7.26	8.38	14.78	486
**8b**	7.77	7.59	14.88	476
**9a**	7.94	10.04	14.90	309
**9b**	6.68	11.29	8.76	330
**10**	6.40	10.39	9.42	435
**11**	4.39	12.46	12.28	316
**12**	7.27	7.09	8.85	265
**13**	12.23	10.85	9.65	735
**14**	39.60	66.09	80.84	>1000

As shown in Table 1, all of the synthesized compounds exhibited good *in vitro* cytotoxicity against all four of the tumor cell lines tested, with IC_50_ values of less than 800 nM in all cases, except for compound **14**. Compound **7** (CA-4E) showed the greatest cytotoxicity of all of the compounds tested against all four cell lines with IC_50_ values in the range of 1 to 180 nM. It is noteworthy that CA-4E showed lower cytotoxicities than those of CA-4. Compound **6** was only weakly cytotoxic compared to CA-4, except against the MCF-7 cells. Compounds **8a**, **8b**, **9a**, **9b**, **10**, **11** and **12** displayed similar cytotoxicities to the parent compound CA-4 against the MDA-MB-231, MCF-7 and A549 cells. However, compounds **13** and **14**, especially **14**, showed much lower cytotoxicities than CA-4, and these lower cytotoxicities were attributed to the size of the 3’-O-substituted carbonic ether moiety, in that the long side chain in **14** most likely prevented the binding of the active moiety to the colchicine site of tubulin. These results therefore suggested that the modification of CA-4 with bulky substituents at the C-3’ position would lead to a reduction in the activity of the inhibitors.

**Figure 3:**
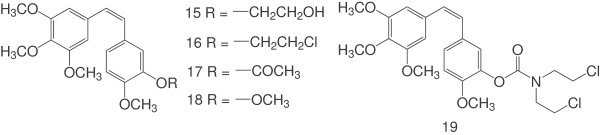
Structures of the CA-4 derivatives.

Several CA-4 derivatives bearing similar substituents to those of CA-4E at the 3’-position of their B-ring, such as a hydroxyethyl (**15**), chloroethyl (**16**), acetyl (**17**), methyl (**18**) or chloroethyl-carbamoyl (**19**) group (Figure 3), were synthesized and evaluated for their antitumor activity. As previously reported, compounds **15**, **16** and **19** exhibited very low levels of cytotoxicity against the tumor cell lines tested (i.e., SK-N-SH, SW1736, NCI-H460, DU-145 and FADU cells), with IC_50_ values in the range of 28 to 4,700 nM, whereas CA-4 exhibited IC_50_ values in the range of 0.26 to 0.76 nM against the same cell lines [13]. Compound **17** displayed similar cytotoxicity to CA-4 against the murine leukemic cell line L1210, with an IC_50_ value of 7 × 10^3^ nM, whereas compound **18** gave an IC_50_ value of 3 × 10^5^ nM against the same cell line [20]. Taken together, these results clearly demonstrate that CA-4E exhibited higher levels of cytotoxicity than CA-4 against all four of the tumor cell lines tested. Further studies would be required, however, to determine whether the additional activity of CA-4E was due to its carbonic acid ester (CA-4E) or decomposition products, and potentially develop a clinical useful anticancer agent.

#### Cell cycle analysis

**Figure 4:**
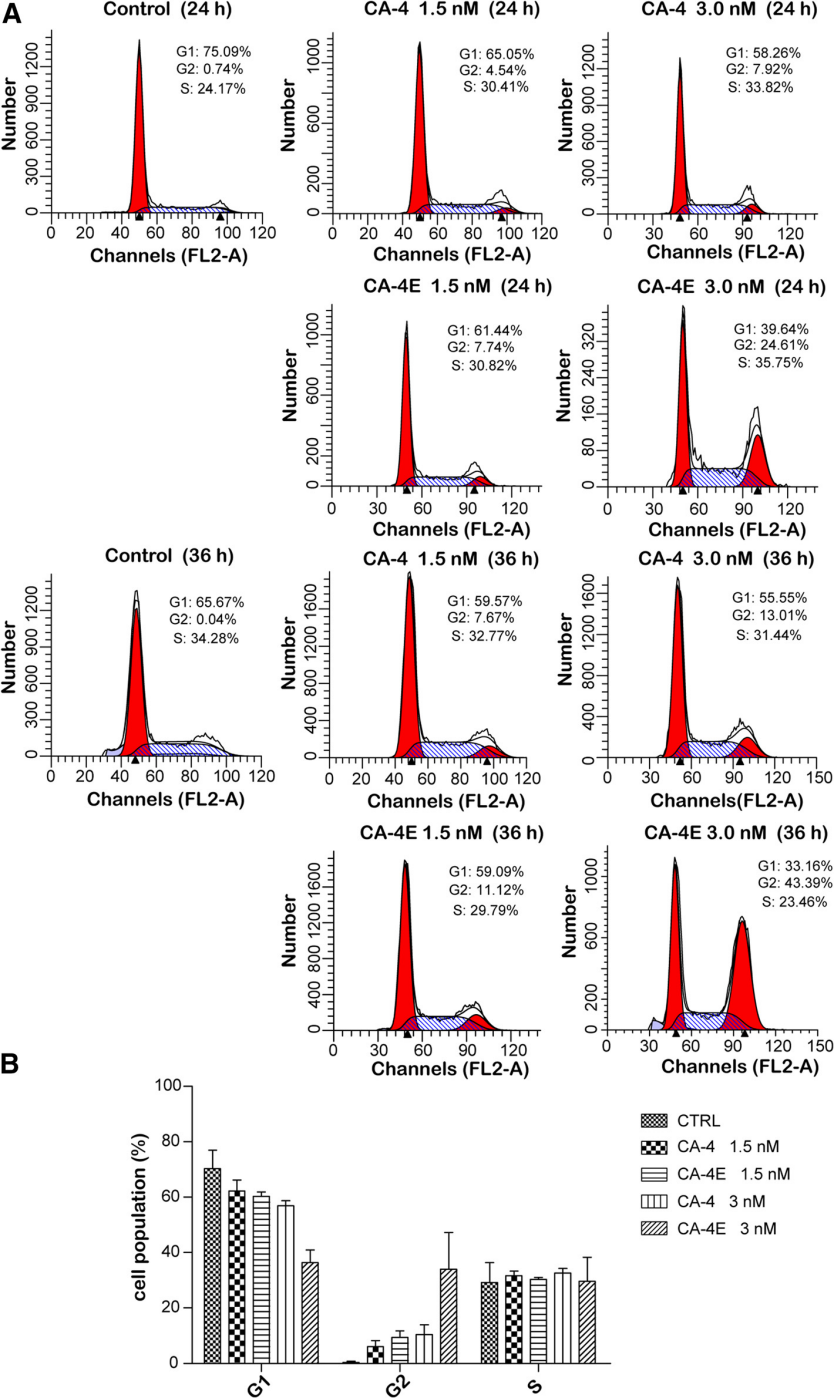
**Effects of CA-4 and CA-4E on the cell cycle distribution of MCF-7 cells.** MCF-7 cells were cultured for 24 and 36 h without any compound (control) and with CA-4 or CA-4E at concentrations of 1.5 and 3 nM, respectively. **(A)** Cycle distribution of the cells was detected by flow cytometry. **(B)** Average population of cells in G1, G2 24 and S phase, respectively.

In light of its remarkable antitumor activity, CA-4E was selected as being the best representative of the synthesized compounds and its antitumor effects on the cell cycle were investigated further and compared to those of CA-4. MCF-7 cells were treated with 1.5 and 3 nM of CA-4 and CA-4E for 24 and 36 h, respectively. The cells were then analyzed by FCM, and the results are shown in Figure 4. The G1 phase frequencies were 75.09, 65.05 and 58.26% after treatment with 0, 1.5, and 3 nM of CA-4 for 24 h, respectively. Furthermore, the treatment of the MCF-7 cells with 0, 1.5, and 3 nM of CA-4E led to G1 phase frequencies of 75.09, 61.44 and 39.64%, respectively. The results illustrated that treatment with CA-4 or CA-4E led to similar reductions in the number of cells in the G1 phase, and the effect of CA-4E was more pronounced, especially at the higher concentration. The frequencies of cells in the G2/M phase following treatment with 0, 1.5, and 3 nM of CA-4 were 0.74, 4.54 and 7.92%, respectively, whereas treatment with the same concentration of CA-4E gave G2/M frequencies of 0.74, 7.74 and 24.61%, respectively. These results indicated that CA-4 and CA-4E both led to a concentration-dependent increase in the number of cells in the G2/M phase, with the effect of CA-4E being more pronounced than that of CA-4, especially at the higher concentration. Similar results were also observed for the cells cultured for 36 h, except the number of cells in the S phase increased after culturing for 24 h, whereas a decrease was observed after culturing for 36 h. These results demonstrated that CA-4E induced the transfer of the cell arrest phase from the G1 phase to the G2/M phase via the S phase. These cell cycle analysis experiments demonstrated that CA-4E induced both time- and dose-dependent G2/M arrest in the same way as CA-4, but more powerfully.

#### Effect on apoptosis

**Figure 5:**
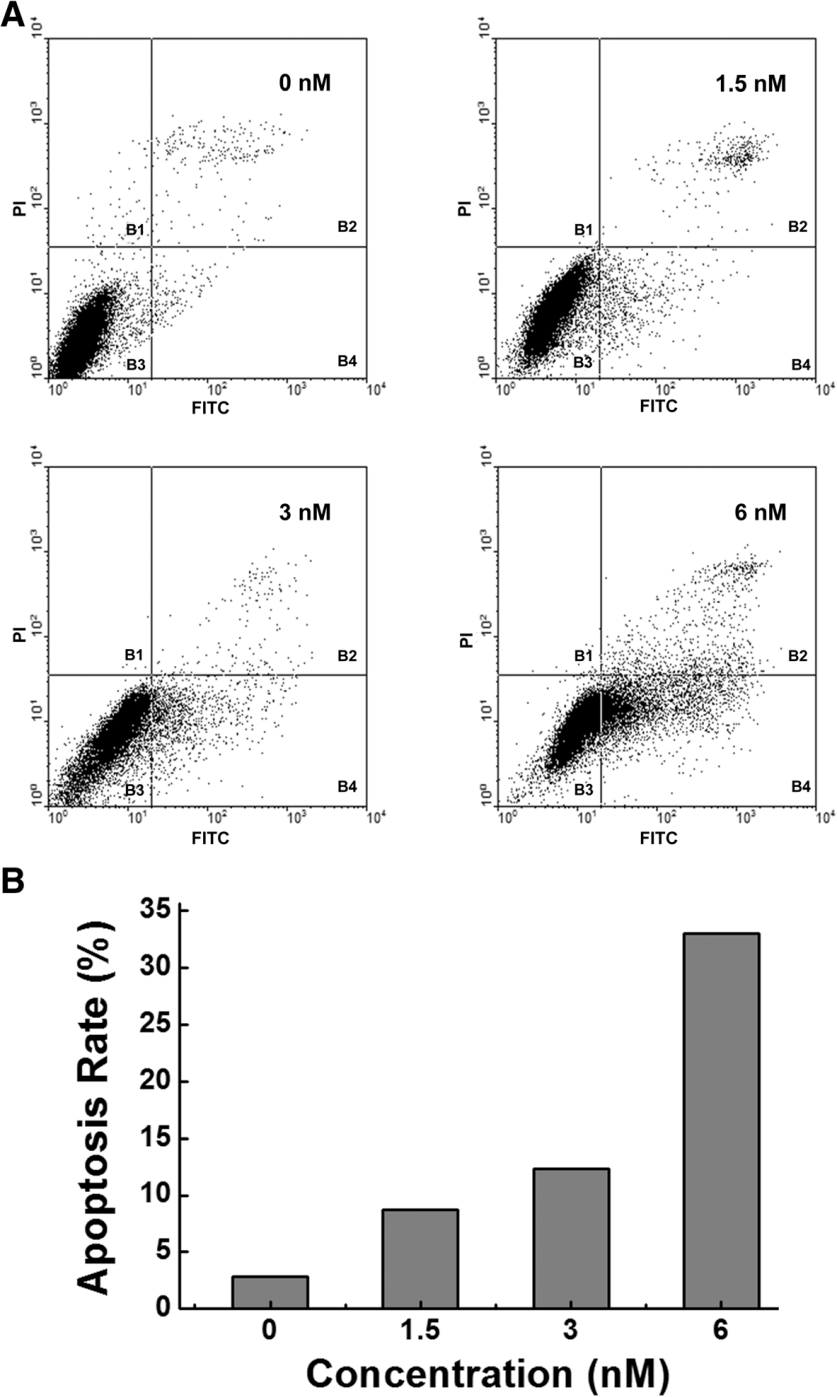
** Apoptotic effect on MCF-7 cell line induced by CA-4E.** MFC-7 cells were incubated in media containing various concentrations of CA-4E (i.e., 0, 1.5, 3 and 6 nM) for 24 h. **(A)** Apoptotic cell scatter plots were constructed by flow cytometry using FITV-Annexin V/PI staining. **(B)** Rate of apoptotic cells at different concentrations of CA-4E.

The effect of CA-4E on the apoptosis of MCF-7 cells was also evaluated. MCF-7 cells were exposed to different concentrations of CA-4E for 24 h, and the FITC-Annexin V/PI double staining method for FCM was used to construct apoptotic cell scatter plots. The results are shown in Figure 5 (A). The apoptotic cell scatter plot was divided into four quadrants, including the B1 quadrant, which represents the damaged cells; B2 quadrant, which represents late apoptotic cells; B3 quadrant representing living cells; and B4 quadrant, which represents early apoptotic cells. The cells in the B2 and B4 quadrants were regarded as apoptotic cells, and were used to evaluate the effect of CA-4E on apoptosis. The total percentages of apoptotic cells were 2.84, 8.74, 12.34 and 33.03% at concentrations of 0, 1.5, 3 and 6 nM, respectively. As shown in Figure 5 (B), the rate of apoptosis increased sharply with increasing CA-4E concentration. This result demonstrated that CA-4E induced cellular apoptosis in a dose-dependent manner.

### Experimental section

#### Chemistry

FCC was performed with 200–300 mesh silica gel (10-40 μm, Qingdao Haiyang Chemical Co. Ltd., Qingdao, China). The reactions were monitored by thin-layer chromatography using silica gel GF_254_ plates (Qingdao Haiyang Chemical Co. Ltd.), and the plates were visualization by UV light. Melting points were measured on a Gallenkamp melting point apparatus (Beijing, China). NMR spectra were recorded on a Bruker Avance 300 MHz NMR spectrometer (Bruker, Billerica, Massachusetts, USA) at 300 (^1^H) and 75 (^13^C) MHz using TMS as an internal reference standard. HRMS were conducted on an LTQ-Qrbitrap XL (Thermo-Fisher, Cambridge, Massachusetts, USA). The experimental procedures for preparation of CA-4 and the target compounds **6**-**14**, as well as copies of ^1^H-NMR and ^13^C-NMR, can be found in Additional file 2.

### Biological activity evaluation

#### Cell culture and antitumor activity

Four human tumor cell lines were tested in the current study, including MDA-MB-231 breast cancer, MCF-7 breast cancer, K562 leukemia, A549 lung cancer cells, which were obtained from the Immune Pharmacological Research Institute at Shandong University. All of the test compounds were dissolved in DMSO at 10 μM, and subsequently diluted to the appropriate concentration prior to the addition to the cells. All four human tumor cell lines were cultured in an RPMI 1640 medium (GIBCO) supplemented with 10% bovine fetal calf serum. The cell lines were maintained at 37°C in a humidified atmosphere containing 5% CO_2_ in an incubator. The cancer cells were seeded in 96-well plates and treated with different concentrations of the synthesized compounds after 6 h of incubation. Five replicate wells were used for each concentration, and the concentration of DMSO used in each case never exceeded 0.1%, so that it would not affect the growth of the cells. The treated cells were incubated for 72 h and 10 μL of the cell proliferation reagent WST-1 was then added to each well. The wells were then incubated at 37°C under 5% CO_2_ in a humidified incubator for 2 h. The absorbance was measured in a microplate reader at 450/630 nm. The IC_50_ values were then calculated according to the percentage of growth in the presence of the test compounds.

#### Cell cycle analysis

For the cell cycle analysis experiments, MCF-7 cells (3 × 10^5^) were seeded in 6-well plates and treated with different concentrations of CA-4 and CA-4E (i.e., 0, 1.5, and 3 nM). The cells were then incubated for 24 and 32 h before being washed twice with ice-cold PBS, harvested, fixed with ice-cold PBS in 75% ethanol and stored at 4°C overnight. The cells were then incubated with RNase A (0.1 mg/mL) at 37°C for 45 min, and then stained with propidium iodide (0.1 mg/mL) for 30 min on ice in the absence of light. The DNA contents of 10,000 events were measured by flow cytometery and the cell cycle profiles were analyzed on the basis of the DNA content histograms [21].

#### Cell apoptosis with FITC-Annexin V/PI double staining

Trypsin without EDTA was used to digest and collect the control group and the cells treated with 1.5 and 3 nM of CA-4 and CA-4E. Flow cytometry was performed according to the manufacturer’s procedure provided with the apoptosis detection kit. The MCF-7 cells were washed twice with PBS and centrifuged at 650 × g for 5 min. A binding buffer suspension (500 μL) was added to the cells followed by 5 μL of the FITC-Annexin V mix, and the resulting mixture was held at 4°C for 20 min. Two and a half microliters of the PI mix was then added to the mixture, and the resulting cell suspension was held at 4°C in the absence of light for 15 min. Flow cytometry was performed using a BD FACS Caliber instrument (BD Biosciences, San Jose, USA).

## Conclusion

In summary, we have successfully synthesized a novel series of CA-4 analogues bearing a 3’-O-substituted carbonic ether moiety and evaluated their antitumor activities against four tumor cell lines using a WST-1-based cytotoxicity assay. The results revealed that all of the synthesized compounds exhibited high levels of antitumor activity, with most of the compounds exhibiting similar levels of bioactivity to CA-4. Compound CA-4E, in particular, showed much higher levels of bioactivity than CA-4, with IC_50_ values in the range of 1 to 180 nM. For this reason, CA-4E was selected as the best representative of the synthesized compounds to investigate the antitumor mechanism of these analogues by assessing the effect of CA-4E on the cell cycle and apoptosis. The cell cycle results demonstrated that CA-4E induced time- and dose-dependent G2/M arrest in the same way as CA-4, although the effect of CA-4E was more pronounced than that of CA-4. Furthermore, the apoptosis data showed that CA-4E induced cellular apoptosis in a dose-dependent manner. Taken together, these results suggest that the anticancer activity of CA-4E is worthy of further study, with CA-4E representing a potential new anticancer drug candidate.

## Competing interests

The authors declare that they have no competing interests.

## Authors’ contributions

The current study represents the outcome of constructive discussions between all of the authors. SL and LH offered the necessary guidance to MM to successful carrying out the synthesis, characterization and bioactivity evaluation experiments. JM assisted with the synthetic work. All of the authors have read and approved the final manuscript.

## Supplementary Material

Additional file 1 **Synthetic route of CA-4 and target compounds 6-14.** This file contains the synthetic route to CA-4 and the novel derivatives 6-14 which were synthesized from 3,4,5-trimethoxyphenylacetic acid (1) and 3-hydroxy-4-methoxybenzaldehyde (2). Click here for file

Additional file 2 **Experimental details of the preparation and data for CA-4 and its derivatives 6-14.** This file includes the experimental procedures CA-4 and target compounds 6-14 and their spectroscopic data, as well as the copies of ^1^H-NMR and ^13^C-NMR. Click here for file
