# Supplementary material for: Synthesis and biological evaluation of Combretastatin A-4 derivatives containing a 3’-O-substituted carbonic ether moiety as potential antitumor agents
Source: Chem Cent J. 2013 Dec 5;7:179. doi: 10.1186/1752-153X-7-179 (PMC3878987; doi:10.1186/1752-153X-7-179)
Supplement: Additional file 1 — Synthetic route of CA-4 and target compounds 6-14. This file contains the synthetic route to CA-4 and the novel derivatives 6-14 which were synthesized from 3,4,5-trimethoxyphenylacetic acid (1) and 3-hydroxy-4-methoxybenzaldehyde (2). [file 1752-153X-7-179-S1.docx]

Additional file 1

**Synthesis and biological evaluation of Combretastatin A-4 derivatives containing a 3′-O-substituted carbonic ether moiety as potential antitumor agents**

Mingyi Ma^a^, Longru Sun^a,^ *, Hongxiang Lou^a^, Mei Ji^a^

^a^ Department of National Products Chemistry, Key Lab of Chemical Biology (MOE), School of Pharmaceutical Sciences, Shandong University, Jinan 250012, P. R. China

* Corresponding author:

Longru Sun

Department of National Products Chemistry, Key Lab of Chemical Biology (MOE), School of Pharmaceutical Sciences, Shandong University, No. 44 West Wenhua Road, Jinan 250012, P.R. China

Tel: +86-531-88382012

Fax: +86-531-88382548

E-mail: sunlr@sdu.edu.cn

**Scheme 1.** Synthetic route of CA-4 and the target compounds **6**-**14**
