# Supplementary material for: Synthesis and biological evaluation of Combretastatin A-4 derivatives containing a 3’-O-substituted carbonic ether moiety as potential antitumor agents
Source: Chem Cent J. 2013 Dec 5;7:179. doi: 10.1186/1752-153X-7-179 (PMC3878987; doi:10.1186/1752-153X-7-179)
Supplement: Additional file 2 — Experimental details of the preparation and data for CA-4 and its derivatives 6-14. This file includes the experimental procedures CA-4 and target compounds 6-14 and their spectroscopic data, as well as the copies of 1H-NMR and 13C-NMR. [file 1752-153X-7-179-S2.docx]

Additional file 2

**Synthesis and biological evaluation of Combretastatin A-4 derivatives containing a 3’-O-substituted carbonic ether moiety as potential antitumor agents**

MingyiMa^a^, LongruSun^a,^ *, HongxiangLou^a^, Mei Ji^a^

^a^Department of National Products Chemistry, Key Lab of Chemical Biology (MOE), School of Pharmaceutical Sciences, Shandong University, Jinan 250012, P. R. China

* Corresponding author:

Longru Sun

Department of National Products Chemistry, Key Lab of Chemical Biology (MOE), School of Pharmaceutical Sciences, Shandong University,No. 44 West Wenhua Road, Jinan 250012, P.R. China

Tel:+86-531-88382012

Fax: +86-531-88382548

E-mail: sunlr@sdu.edu.cn

***Synthesis of CA-4***

A mixture of 3,4,5-trimethoxyphenylacetic acid (13.56 g, 60 mmol), 3-hydroxy-4-methoxybenzaldehyde (10.94 g, 72 mmol), triethyl-amine (10 mL, 71.88 mmol), and acetic anhydride (20 mL，211.76 mmol) were heated at 140 °C for 3 h at reflux. The mixture, concentrated in vacuum, was added with 1N hydrochloric acid (30 mL) and stirred overnight. Yellow solid obtained was added with [ethanol](app:ds:ethanol) (40 mL) and sodium hydroxide (6 g, 150 mmol) and stirred overnight at room temperature. The [ethanol](app:ds:ethanol) [solution](app:ds:solution) was added 1N hydrochloric acid to reach a pH value of 2-3, and slight yellow solid was filtered off. The solid was washed with water and collected by vacuum suction filtration. The slight yellow solid was added to powdered copper (12 g, 187.6 mmol) in quinoline (120 mL), and the resulting mixture was heated at 220 °C for 2 h. Upon cooling, ether was added, and the copper was filtered off. The filtrate was washed with 1N hydrochloric acid (100 mL), saturated sodium bicarbonate solution (100 mL), tri-distilled water (100 mL), dried with MgSO_4_, and concentrated in vacuum. Flash column chromatography (SiO_2_, petrol : EtOAc = 7:3) and recrystallization from [ethyl](app:ds:ethyl) [acetate](app:ds:acetate) afforded desired CA-4 in 55% yield.

***General procedure for synthesis of target compounds 6-14***

A mixture of CA-4 (0.158 g, 0.5 mmol), anhydrous CH_2_Cl_2_ (10 mL) and pyridine (1 mL, 12.5 mmol) was stirred for 5 min at 0 °C under nitrogen. Alkyl chloroformic ester (2 mmol) was added drop by drop. The mixture was continuously stirred at 0 °C for 30 min and then for 12 h at room temperature under nitrogen. Target compounds 6-14 were purified by column chromatography, and their structures were confirmed by ^1^H-NMR, ^13^C-NMR and HRMS.

**Data for CA-4**

Pale yellow crystalline solid. Yield: 55%.m.p. 115-117 °C. ^1^H-NMR (300 MHz, CDCl_3_) *δ*: 6.93 (d, 1H, *J* = 2.1 Hz, H-2’), 6.80 (dd, 1H, *J* = 8.4, 2.1 Hz, H-6’), 6.74 (d, 1H, *J* = 8.4 Hz, H-5’), 6.53 (s, 2H, H-2, H-6), 6.48 (d, 1H, *J* = 12.0 Hz, H-a or H-a’), 6.40 (d, 1H, *J* = 12.0 Hz, H-a’ or H-a), 3.87 (s, 3H), 3.85 (s, 3H), 3.70 (s, 6H). MS (ESI) *m*/*z*: 317.1 [M+1]^+^.

**Data for compounds 6-14**

***(Z)-2-methoxy-5-(3, 4, 5-trimethoxystyryl)phenyl methyl carbonic ester (6)***

Pale yellow syrup, yield: 78%. ^1^H-NMR (300 MHz, CDCl_3_) *δ*: 7.13 (dd, 1H, *J* = 8.4, 2.1 Hz, H-6’), 7.10 (d, 1H, *J* = 2.1 Hz, H-2’), 6.87 (d, 1H, *J* = 8.4 Hz, H-5’), 6.49 (s, 2H, H-2, H-6), 6.46 (s, 2H, H-a, H-a’), 3.86 (s, 3H, OCH_3_), 3.84 (s, 3H, OCH_3_), 3.82 (s, 3H, OCH_3_), 3.70 (s, 6H, OCH_3_×2); ^13^C-NMR (75 MHz, CDCl_3_) *δ*: 153.8, 153.0, 150.3, 139.8, 137.3, 132.3, 130.1, 129.7, 128.4, 128.0, 122.8, 112.2, 105.9, 60.9, 56.0, 55.9, 55.4; HRMS (ESI) *m/z*: calcd for C_20_H_22_O_7_Na (M+Na) 297.1263, found 297.1274 [M+Na]^+^.


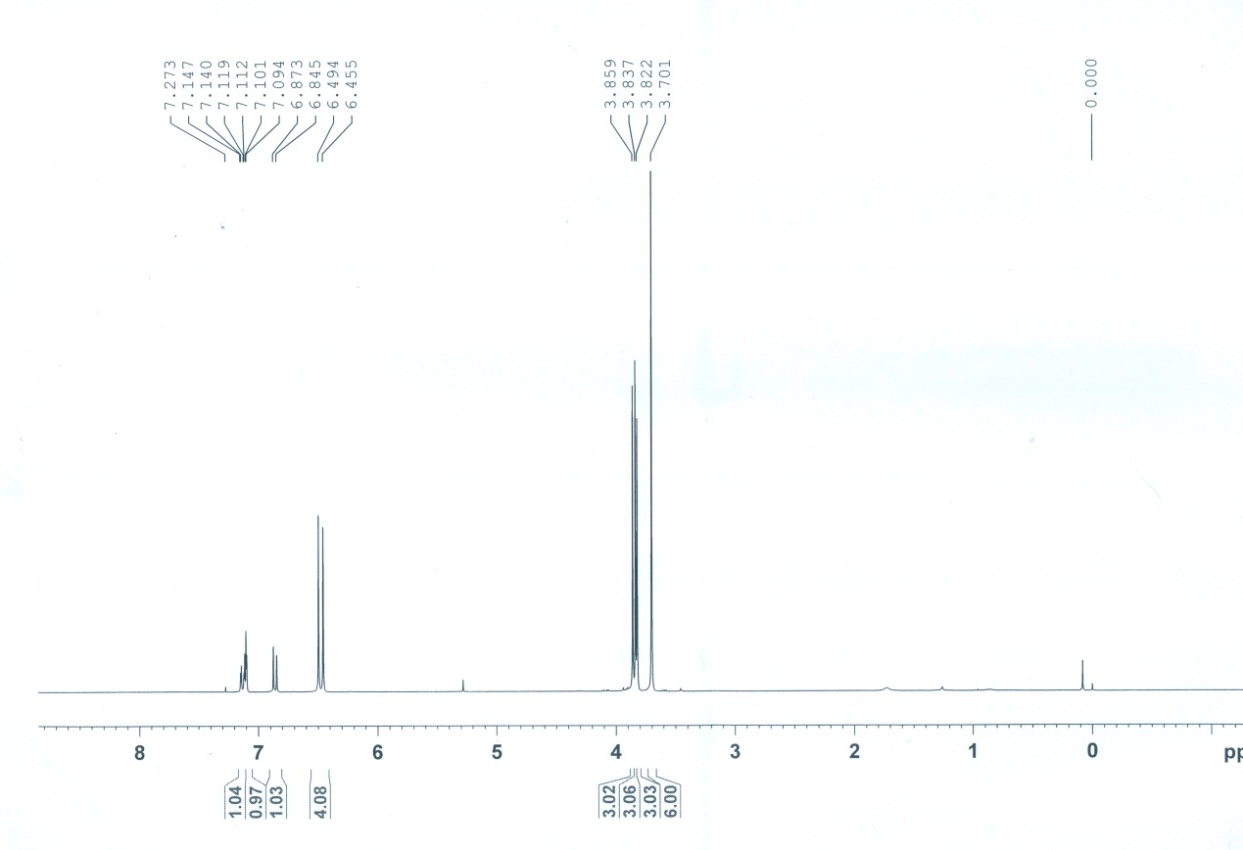


**^1^H NMR for compound 6**


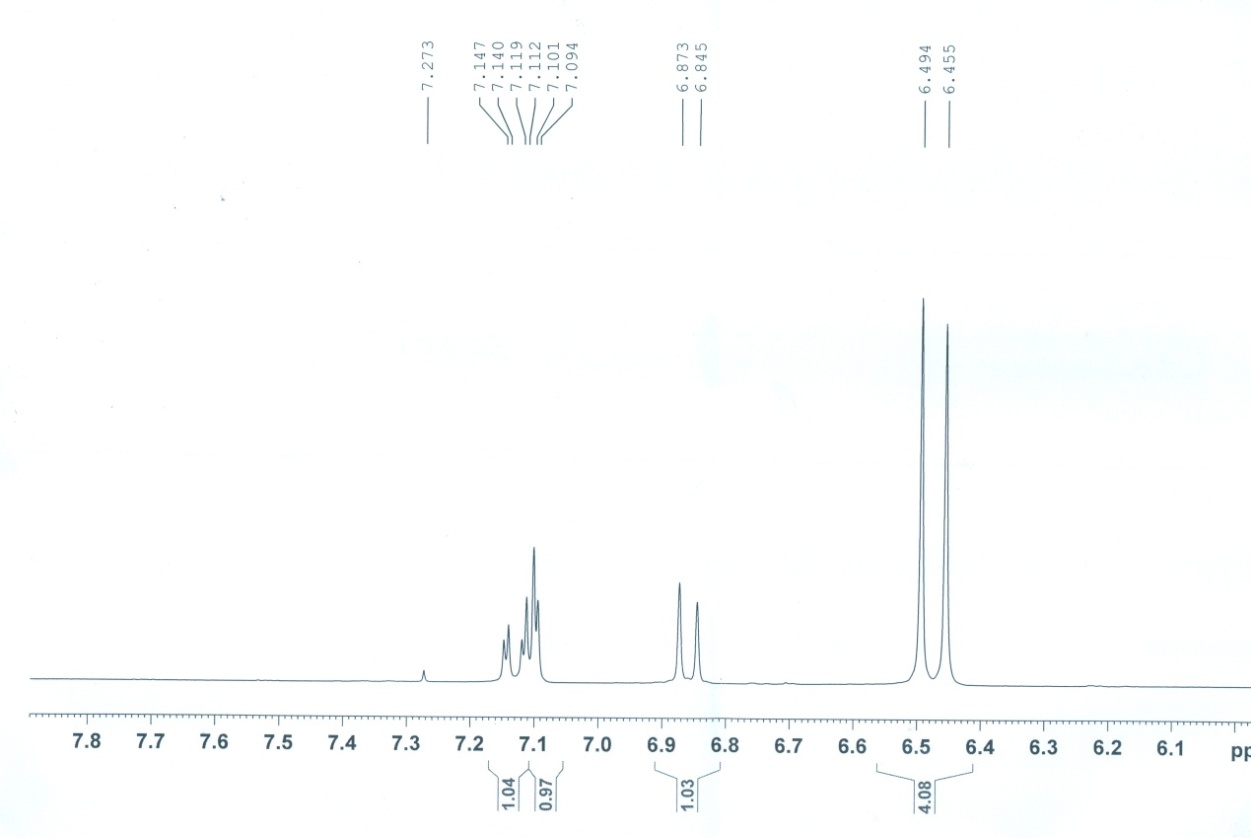


**Partial ^1^H NMR for compound 6**


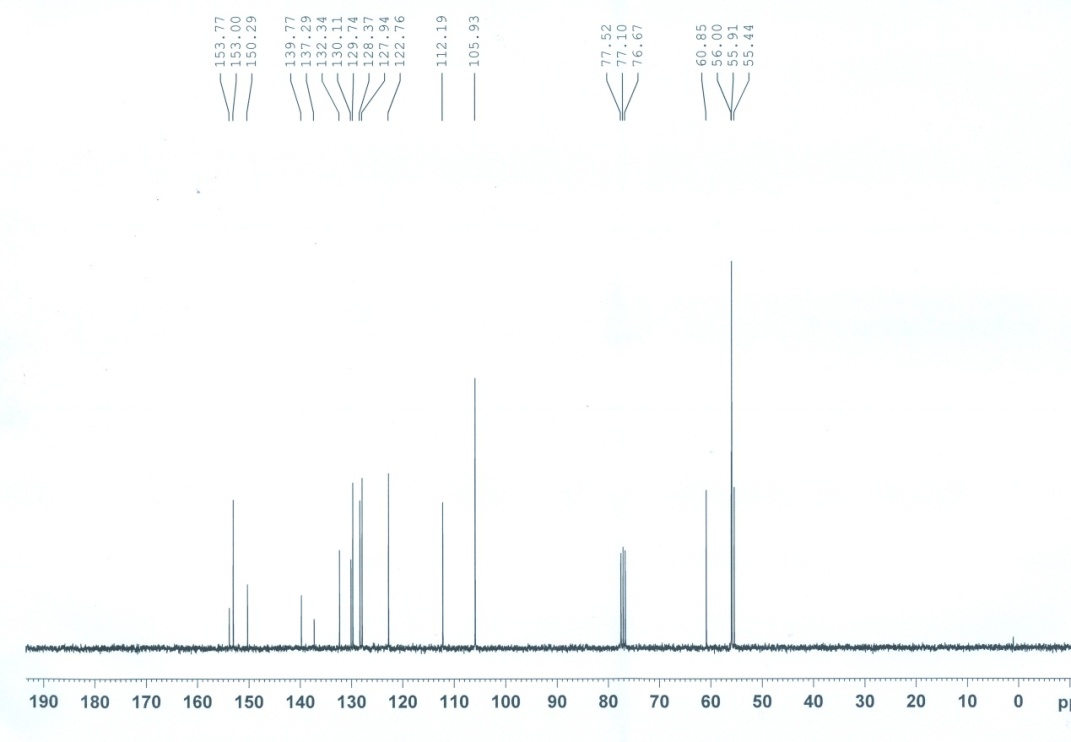


**^13^C NMR for compound 6**

***(Z)-2-methoxy-5-(3, 4, 5-trimethoxystyryl)phenyl ethyl carbonic ester (7)***

Pale yellow syrup, yield: 75%. ^1^H-NMR (300 MHz, CDCl_3_) *δ*: 7.10-7.13 (m, 2H, H-2’, H-6’), 6.86 (d, 1H, *J* = 9.0Hz, H-5’), 6.50 (s, 2H, H-2, H-6), 6.45 (s, 2H, H-a, H-a’), 4.27 (q, 2H, *J* = 7.2Hz, OCH_2_), 3.83 (s, 3H, OCH_3_), 3.81 (s, 3H, OCH_3_), 3.69 (s, 6H, OCH_3_×2), 1.34 (t, 3H, *J* = 7.2Hz, CH_3_); ^13^C-NMR (75 MHz, CDCl_3_) *δ*: 153.1, 153.0, 150.3, 139.8, 137.3, 132.3, 130.1, 129.7, 128.4, 127.9, 122.7, 112.2, 105.9, 64.8, 60.8, 55.9, 55.8, 14.1; HRMS (ESI) *m/z*: calcd for C_21_H_24_O_7_Na (M+Na)411.1420, found 411.1429 [M+Na]^+^.

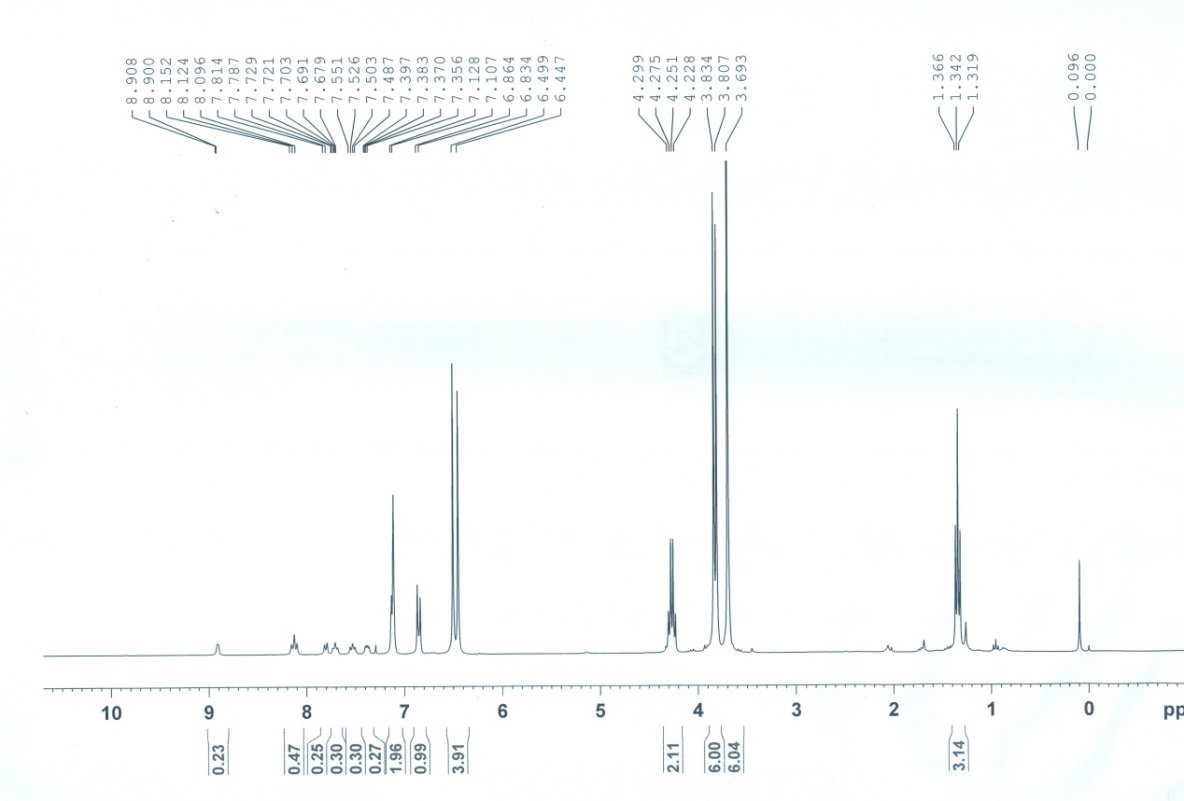


**^1^H NMR for compound 7**


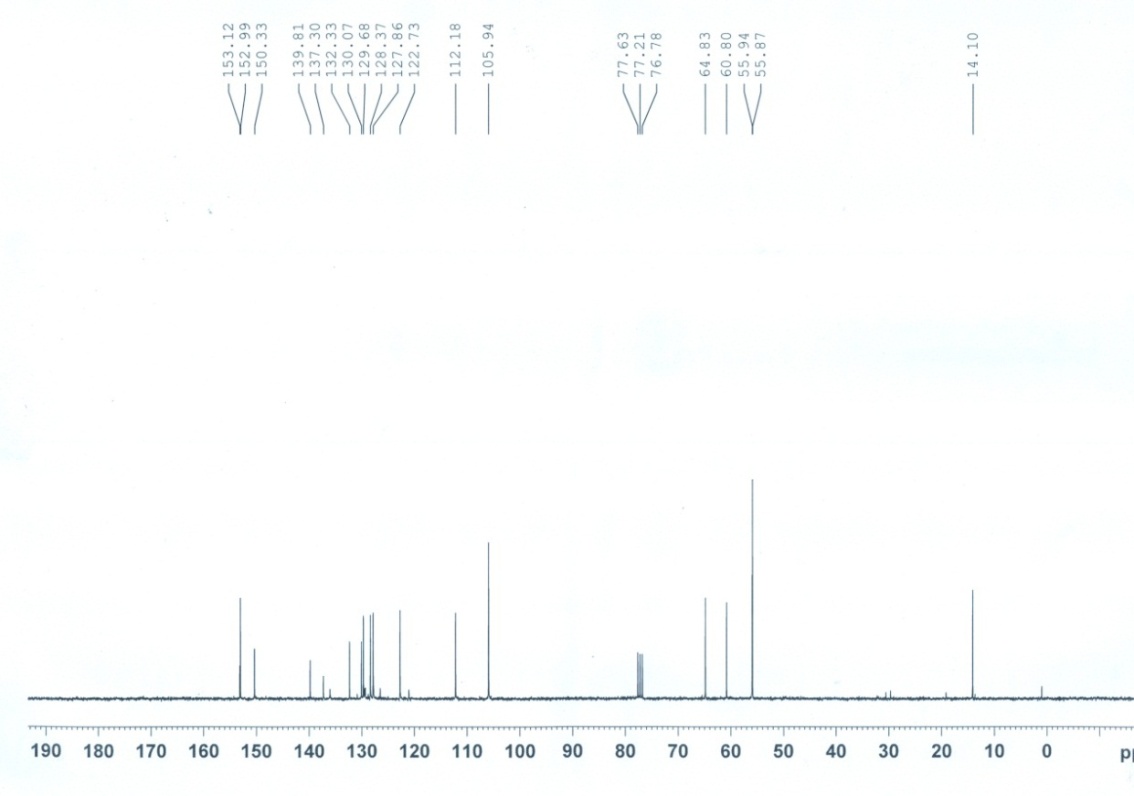


**^13^C NMR for compound 7**

***(Z)-2-methoxy-5-(3, 4, 5-trimethoxystyryl)phenyl propyl carbonic ester (8a)***

Pale yellow syrup, yield:72%.^1^H-NMR (300 MHz, CDCl_3_) *δ*: 7.01-7.15 (m, 2H, H-2’, H-6’), 6.86 (d, 1H, *J* = 9Hz, H-5’), 6.50 (s, 2H, H-2, H-6), 6.45 (s, 2H, H-a, H-a’), 4.18 (t, 2H, *J* = 6.9Hz, OCH_2_), 3.84 (s, 3H, OCH_3_), 3.82 (s, 3H, OCH_3_), 3.70 (s, 6H, OCH_3_×2), 1.75 (m, 2H, CH_2_), 0.99 (t, 3H, *J* = 7.8Hz, CH_3_); ^13^C-NMR (75 MHz, CDCl_3_) *δ*: 153.3, 153.0, 150.3, 139.8, 137.3, 132.4, 130.1, 129.7, 128.4, 127.9, 122.8, 112.2, 105.9, 70.4, 60.8, 56.0, 55.9, 22.0, 10.1; HRMS (ESI) *m/z*: calcd for C_22_H_26_O_7_Na (M+Na)425.1577, found 425.1591 [M+Na]^+^.

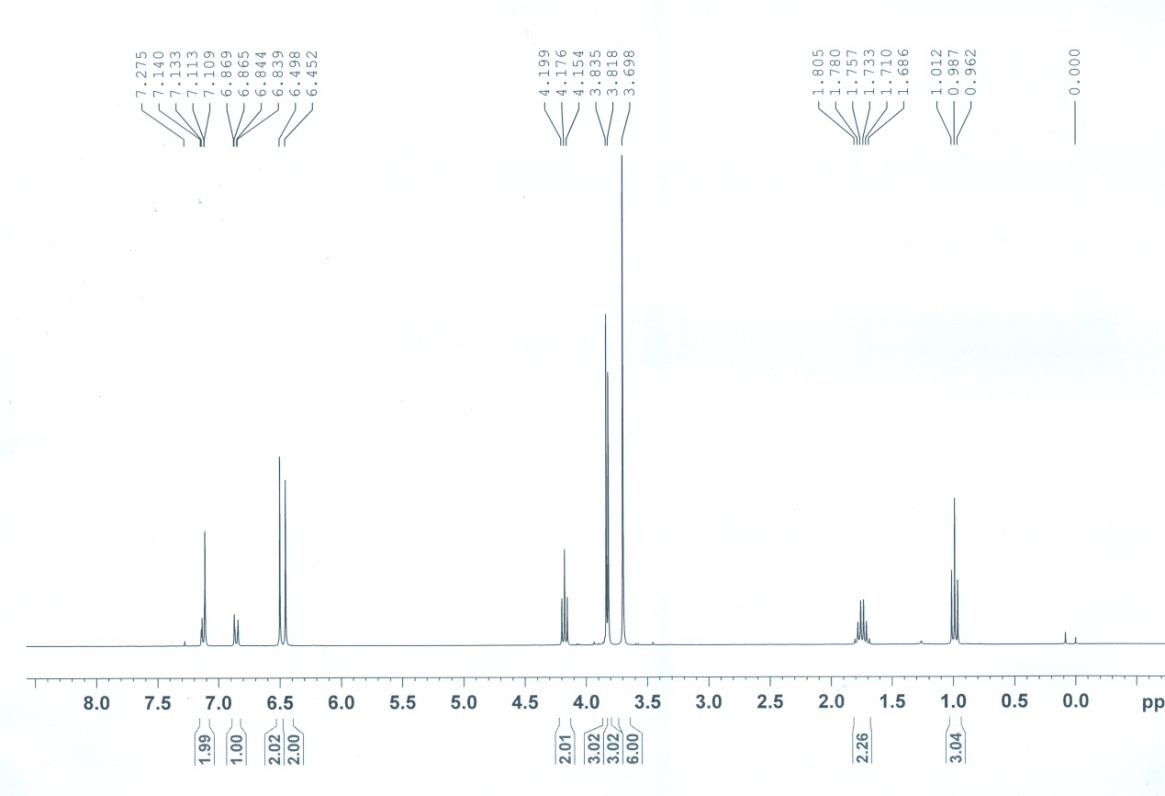


**^1^H NMR for compound 8a**


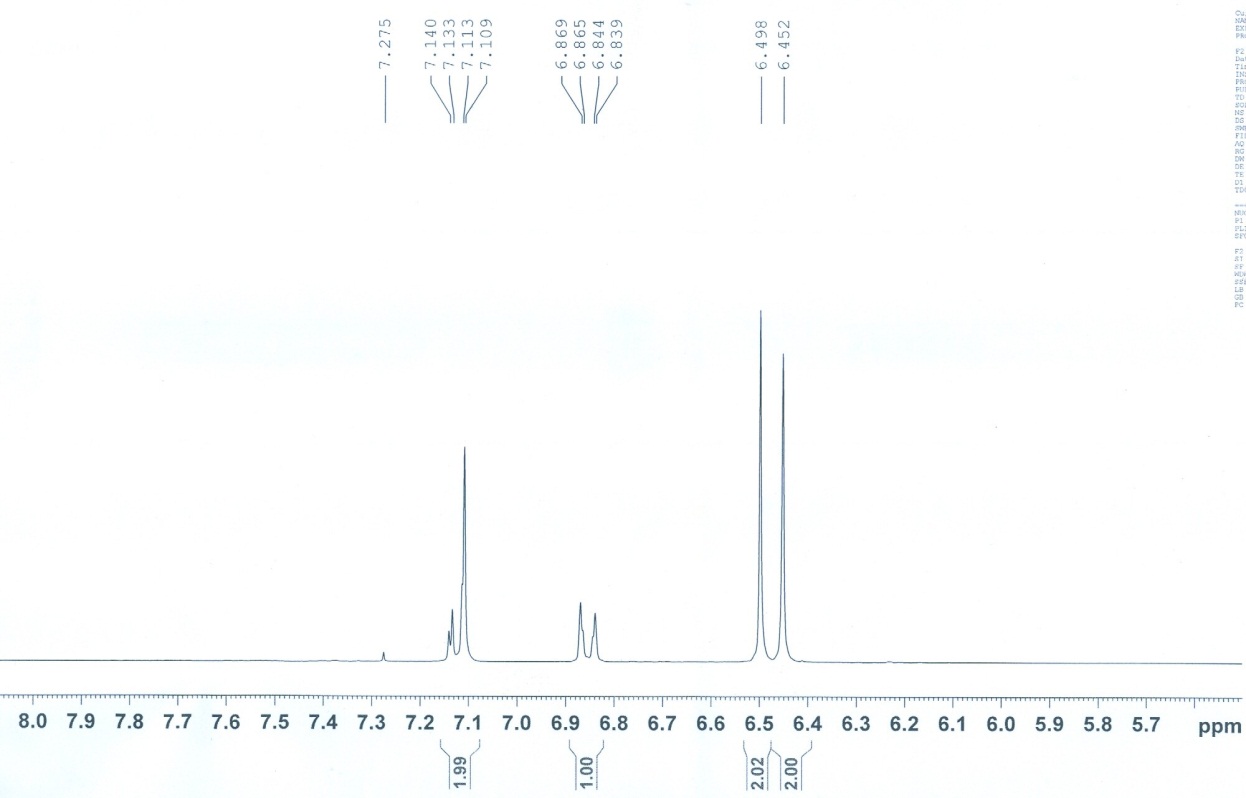


**Partial ^1^H NMR for compound 8a**


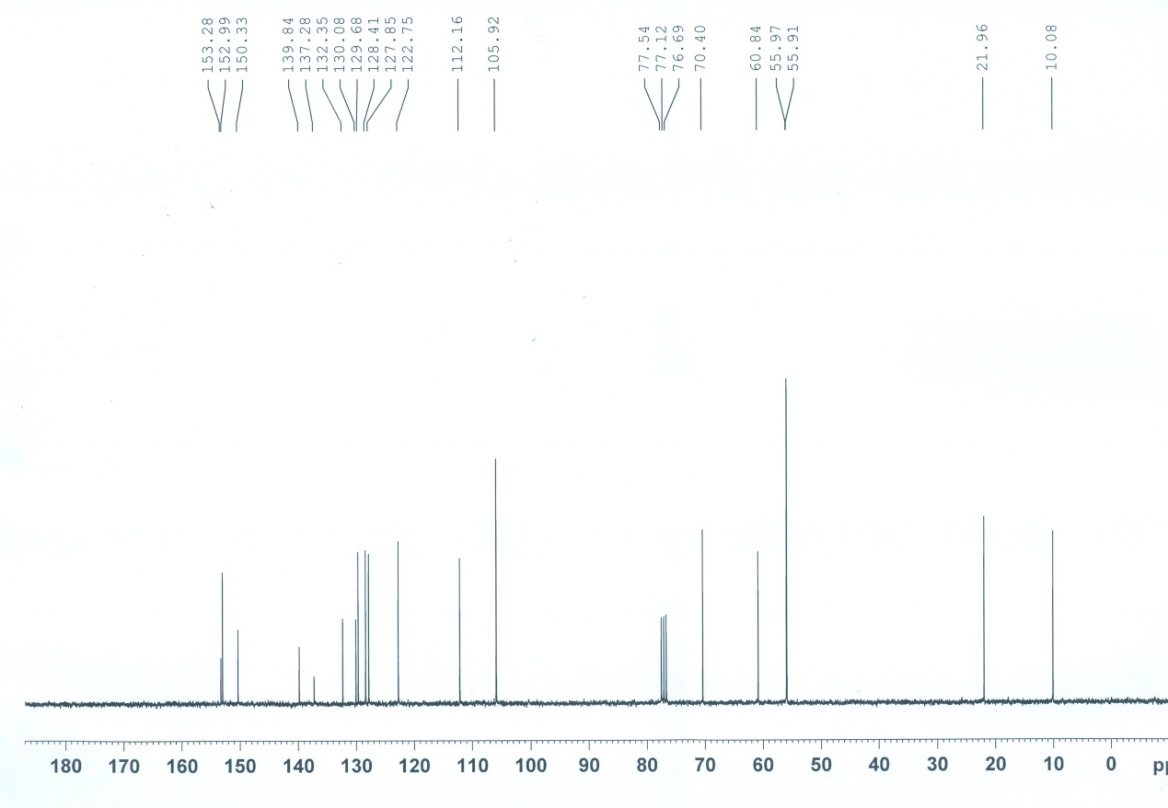


**^13^C NMR for compound 8a**

***(Z)-2-methoxy-5-(3, 4, 5-trimethoxystyryl)phenyl isopropyl carbonic ester (8b)***

Pale yellow syrup, yield:74%.^1^H-NMR (300 MHz, CDCl_3_) *δ*: 7.05-7.14 (m, 2H, H-2’, H-6’), 6.85 (d, 1H, *J* = 9.0Hz, H-5’), 6.50 (s, 2H, H-2, H-6), 6.45 (s, 2H, H-a, H-a’), 4.92 (m, 1H, OCH), 3.83 (s, 3H, OCH_3_), 3.82 (s, 3H, OCH_3_), 3.70 (s, 6H, OCH_3_×2), 1.35 (d, 6H, *J* = 6.3Hz, CH_3_×2);^13^C-NMR (75 MHz, CDCl_3_) *δ*: 153.0, 152.7, 150.4, 139.9, 137.3, 132.4, 130.1, 129.6, 128.4, 127.8, 122.7, 112.2, 105.9, 73.1, 60.8, 56.0, 55.9, 21.6; HRMS (ESI) *m/z*: calcd for C_22_H_26_O_7_Na (M+Na) 425.1577, found 425.1590 [M+Na]^+^.

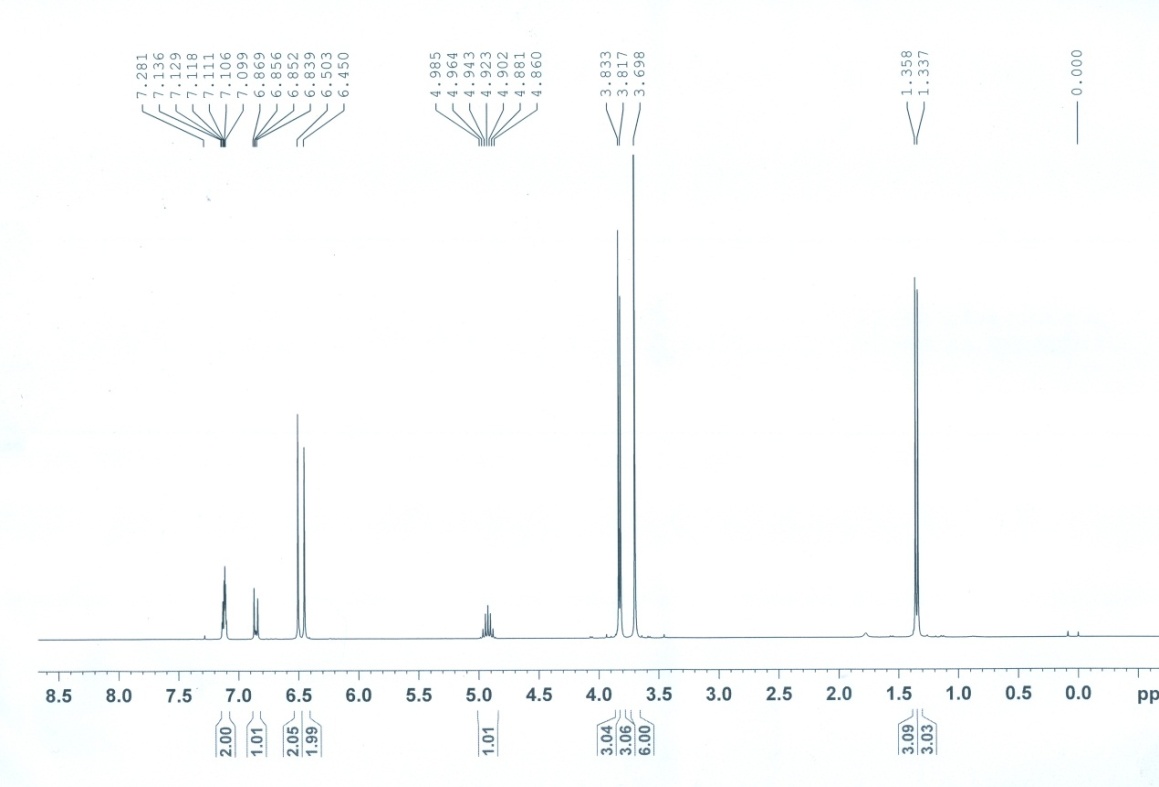


**^1^H NMR for compound 8b**


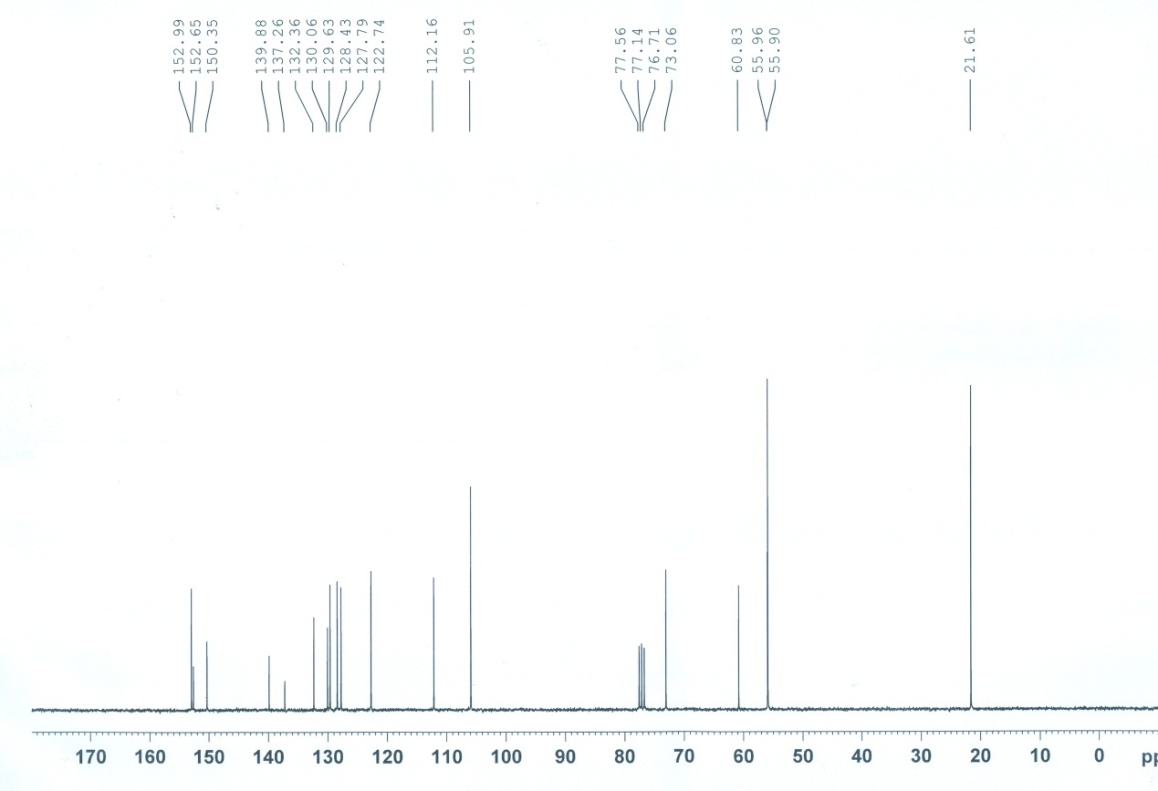


**^13^C NMR for compound 8b**

***(Z)-2-methoxy-5-(3, 4, 5-trimethoxystyryl)phenyl butyl carbonic ester (9a)***

Pale yellow syrup, yield:75%.^1^H-NMR (300 MHz, CDCl_3_) *δ*: 7.10-7.15 (m, 2H, H-2’, H-6’), 6.85 (d, 1H, *J* = 8.1Hz, H-5’), 6.50 (s, 2H, H-2, H-6), 6.45 (s, 2H, H-a, H-a’), 4.22 (t, 2H, *J* = 6.6Hz, OCH_2_), 3.84 (s, 3H, OCH_3_), 3.82 (s, 3H, OCH_3_), 3.70 (s, 6H, OCH_3_×2), 1.72 (m, 2H, CH_2_), 1.44 (m, 2H, CH_2_), 0.96 (t, 3H, *J* = 7.2Hz, CH_3_); ^13^C-NMR (75 MHz, CDCl_3_) *δ*: 153.3, 153.0, 150.3, 139.9, 137.3, 132.4, 130.1, 129.7, 128.4, 127.8, 122.8, 112.2, 105.9, 68.7, 60.9, 56.0, 55.9, 30.6, 18.8, 13.6; HRMS (ESI) *m/z*: calcd for C_23_H_28_O_7_Na (M+Na) 439.1733, found439.1745 [M+Na]^+^.

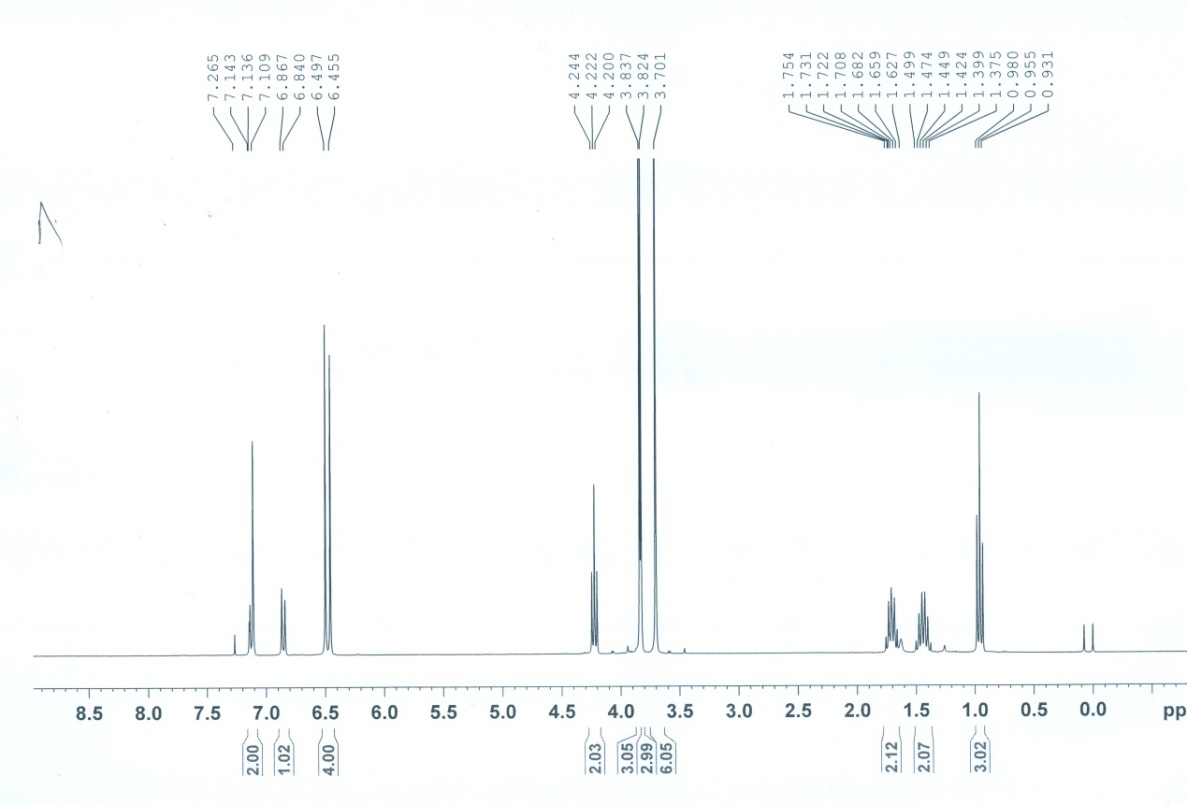


**^1^H NMR for compound 9a**


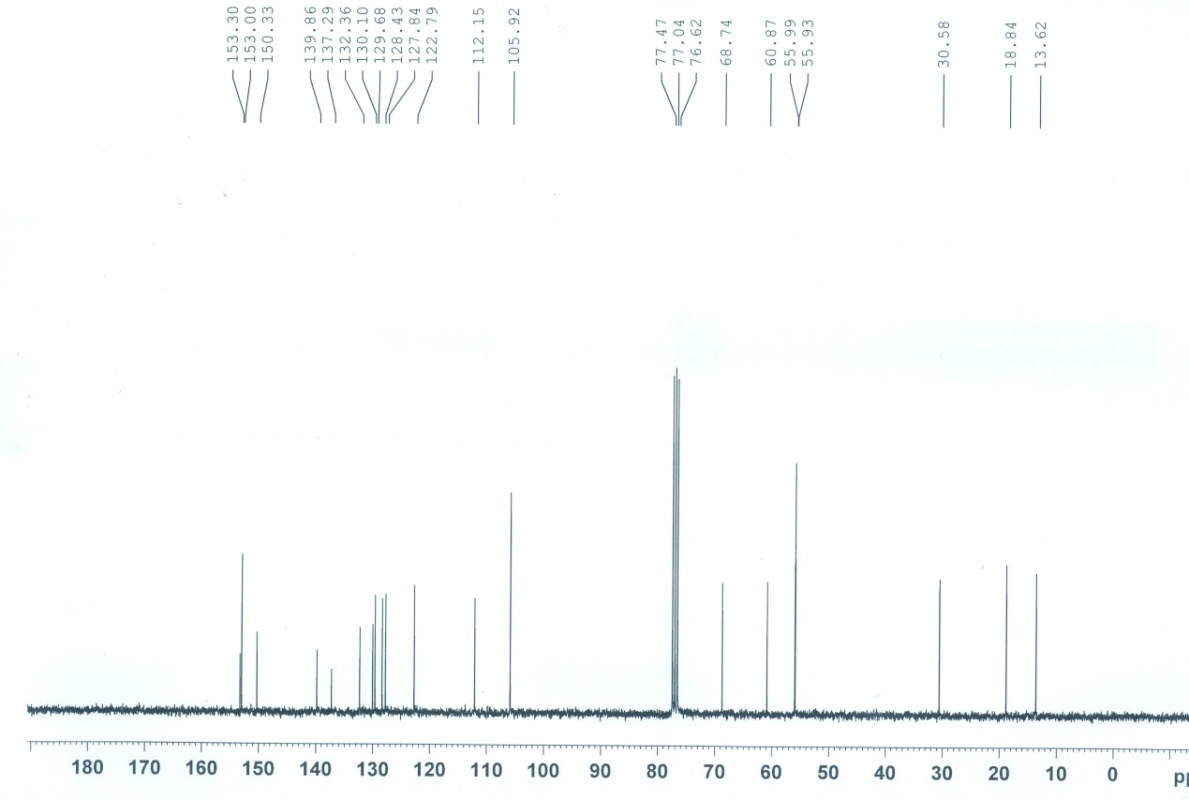


**^13^C NMR for compound 9a**

***(Z)-2-methoxy-5-(3, 4, 5-trimethoxystyryl)phenyl isobutyl carbonic ester (9b)***

Pale yellow syrup, yield:77%. ^1^H-NMR (300 MHz, CDCl_3_) *δ*: 7.09-7.15 (m, 2H, H-2’, H-6’), 6.86 (d, 1H, *J* = 8.4Hz, H-5’), 6.50 (s, 2H, H-2, H-6), 6.46 (s, 2H, H-a, H-a’), 4.01 (d, 2H, *J* = 6.6Hz, OCH_2_), 3.84 (s, 3H, OCH_3_), 3.82 (s, 3H, OCH_3_), 3.70 (s, 6H, OCH_3_×2), 2.03 (m, 1H, CH), 0.98 (d, 6H, *J* = 6.6Hz, CH_3_×2); ^13^C-NMR (75 MHz, CDCl_3_) *δ*: 153.0, 150.3, 139.9, 137.3 132.4, 130.1, 129.7, 128.4, 127.8, 122.8, 112.1, 105.9, 60.9, 56.0, 55.9, 27.8, 18.8; HRMS (ESI) *m/z*: calcd for C_23_H_28_O_7_Na (M+Na)439.1733, found439.1745 [M+Na]^+^.

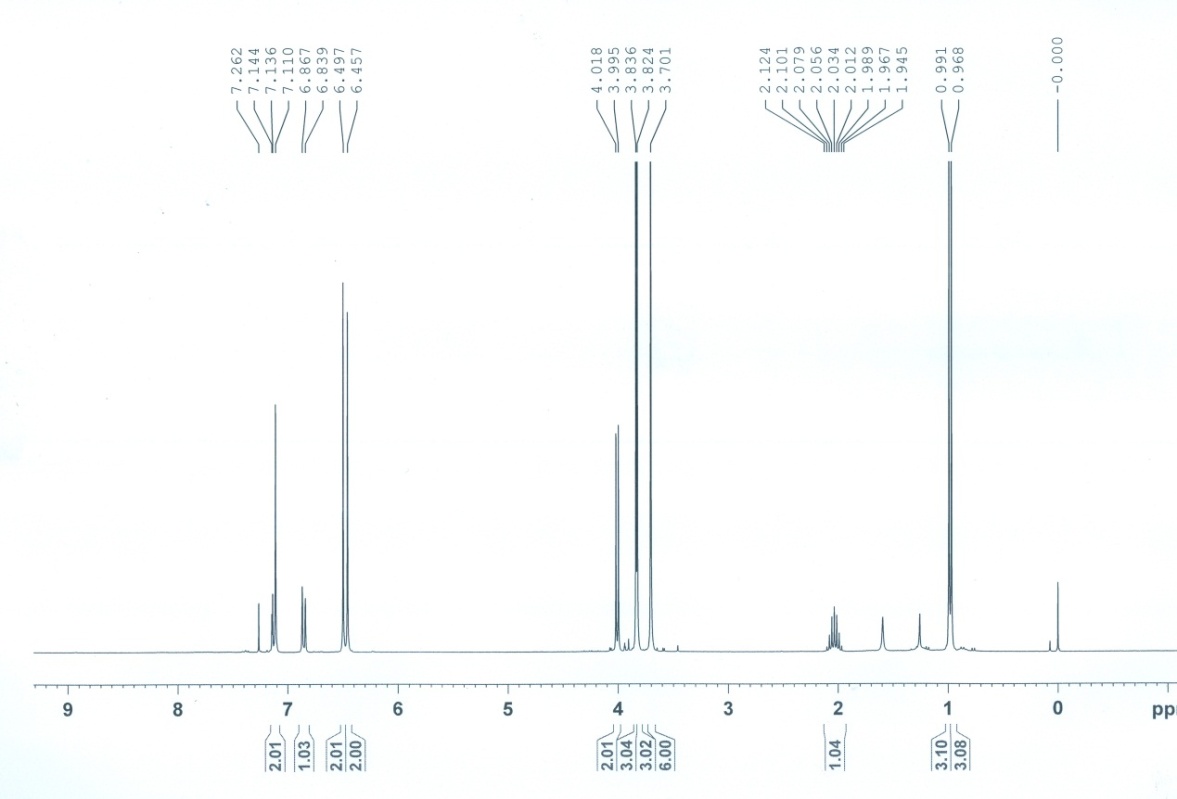


**^1^H NMR for compound 9b**


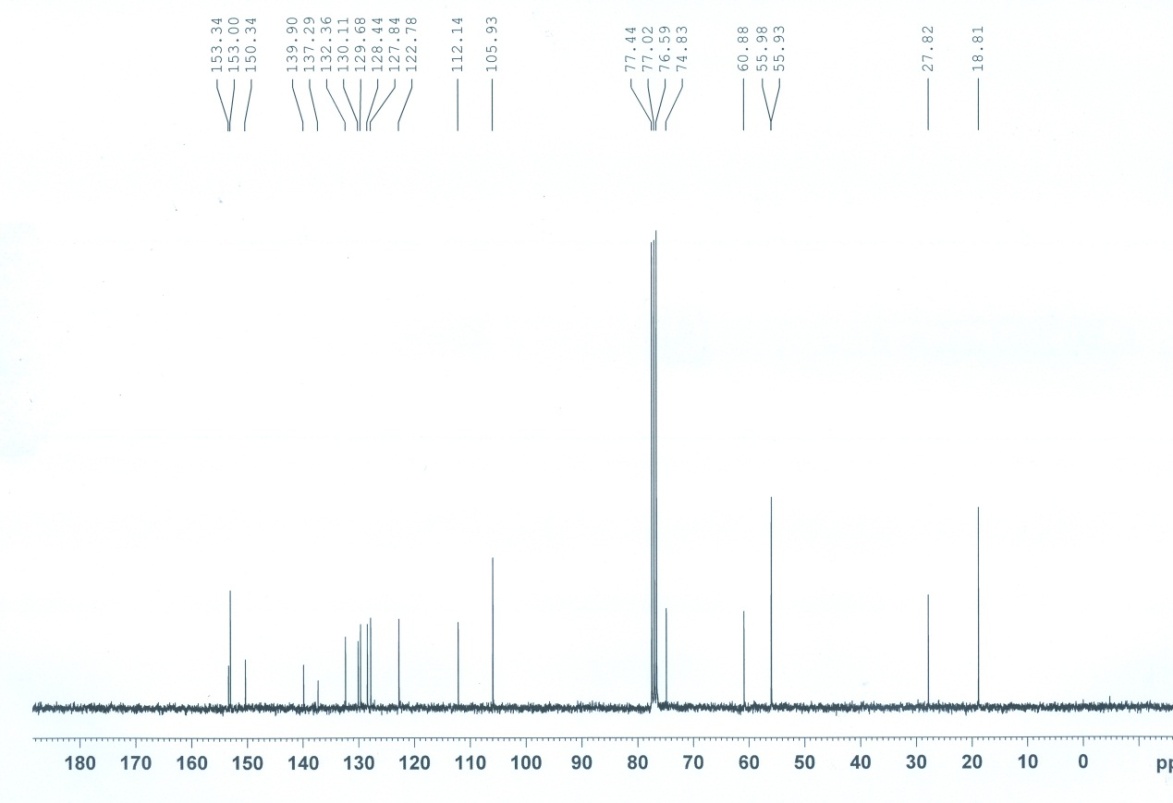


**^13^C NMR for compound 9b**

***(Z)-2-methoxy-5-(3, 4, 5-trimethoxystyryl)phenyl amyl carbonic ester (10)***

Pale yellow syrup, yield: 78%. ^1^H-NMR (300 MHz, CDCl_3_) *δ*: 7.08-7.13 (m, 2H, H-2’, H-6’), 6.86 (d, 1H, *J* = 8.7Hz, H-5’), 6.48 (s, 2H, H-2, H-6), 6.44 (s, 2H, H-a, H-a’), 4.20 (t, 2H, *J* = 6.9Hz, OCH_2_), 3.82 (s, 3H, OCH_3_), 3.81 (s, 3H, OCH_3_), 3.69 (s, 6H, OCH_3_×2), 1.69 (m, 2H, CH_2_), 1.31-1.42 (m, 4H CH_2_×2), 0.90 (t, 3H, *J* = 6.9Hz, CH_3_); ^13^C-NMR (75 MHz, CDCl_3_) *δ*: 153.3, 153.0, 150.3, 139.9, 137.3, 132.3, 130.1, 129.7, 128.4, 127.8, 122.8, 112.2, 105.9, 69.0, 60.8, 56.0, 55.9, 28.2, 27.7, 22.2, 13.9; HRMS (ESI) *m/z*: calcd for C_24_H_30_O_7_Na (M+Na)453.1890, found453.1903 [M+Na]^+^.

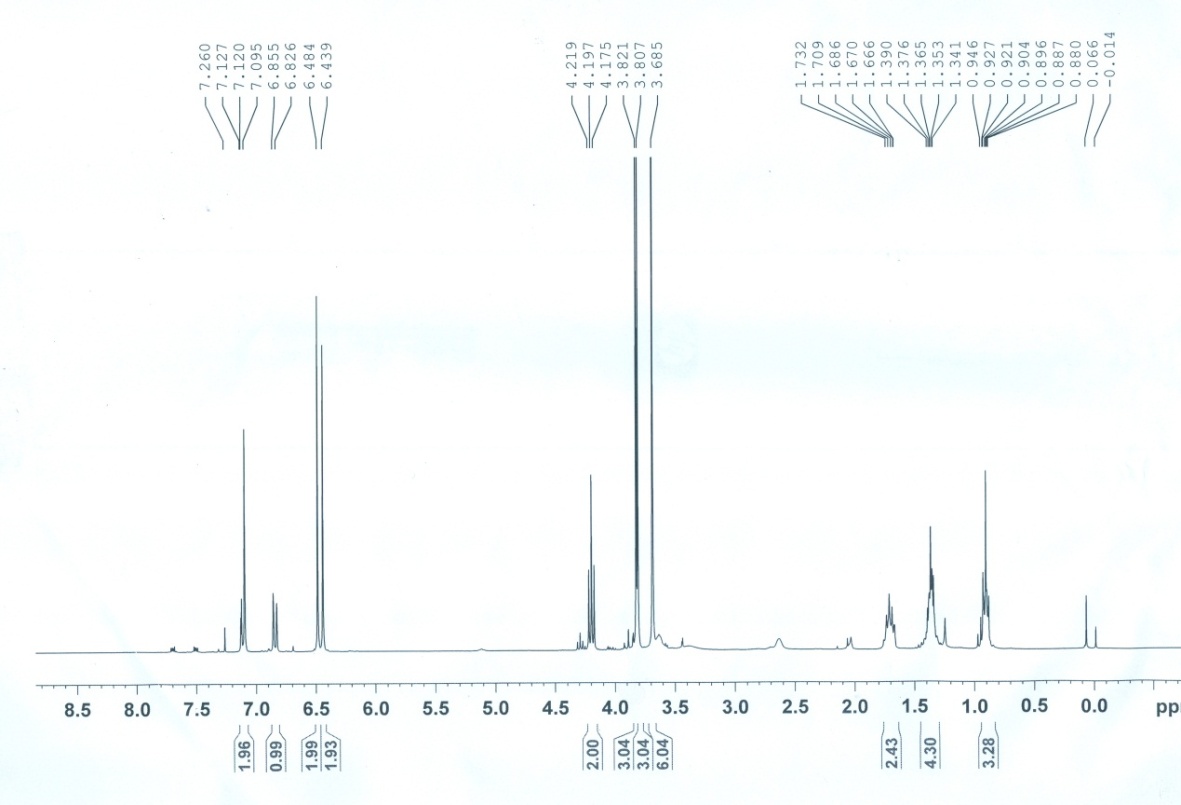


**^1^H NMR for compound 10**


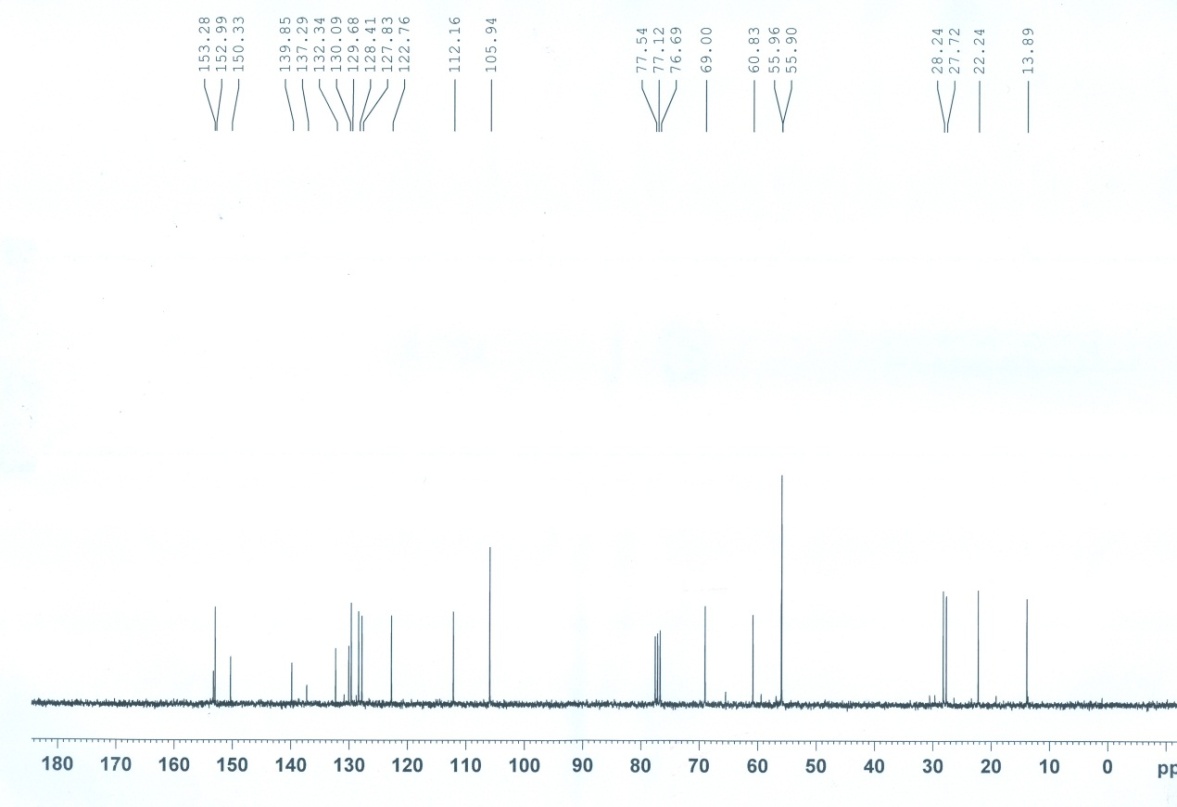


**^13^C NMR for compound 10**

***(Z)-2-methoxy-5-(3, 4, 5-trimethoxystyryl)phenylphenyl carbonic ester (11)***

Pale yellow syrup, yield:76%.^1^H-NMR (300 MHz, CDCl_3_) *δ*: 7.34-7.42 (m, 2H, H-3’’, H-5’’), 7.18-7.27 (m, 4H, H-2’, H-2’’, H-4’’, H-6’’), 7.15 (dd, 1H, *J* = 8.4, 2.1Hz, H-6’), 6.88 (d, 1H, *J* = 8.4Hz, H-5’), 6.50 (s, 2H, H-2, H-6), 6.47 (s, 2H, H-a, H-a’), 3.86 (s, 3H, OCH_3_), 3.83 (s, 3H, OCH_3_), 3.68 (s, 6H, OCH_3_×2); ^13^C-NMR (75 MHz, CDCl_3_) *δ*: 153.1, 151.5, 151.2, 150.2, 139.8, 137.4, 132.3, 130.2, 129.9, 129.5, 128.3, 128.2, 126.2, 122.6, 120.8, 112.3, 106.0, 60.9, 56.1, 55.9; HRMS (ESI) *m/z*: calcd for C_25_H_24_O_7_Na (M+Na)459.1420, found459.1432 [M+Na]^+^.

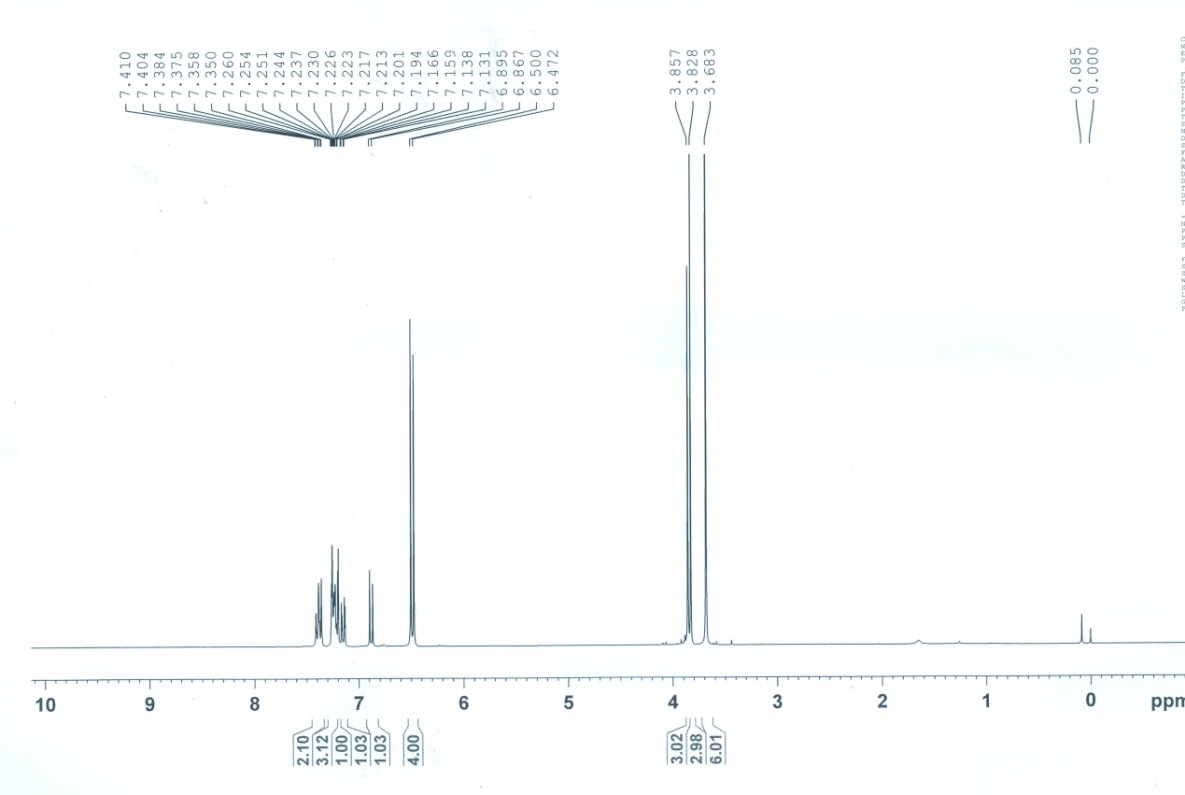


**^1^H NMR for compound 11**


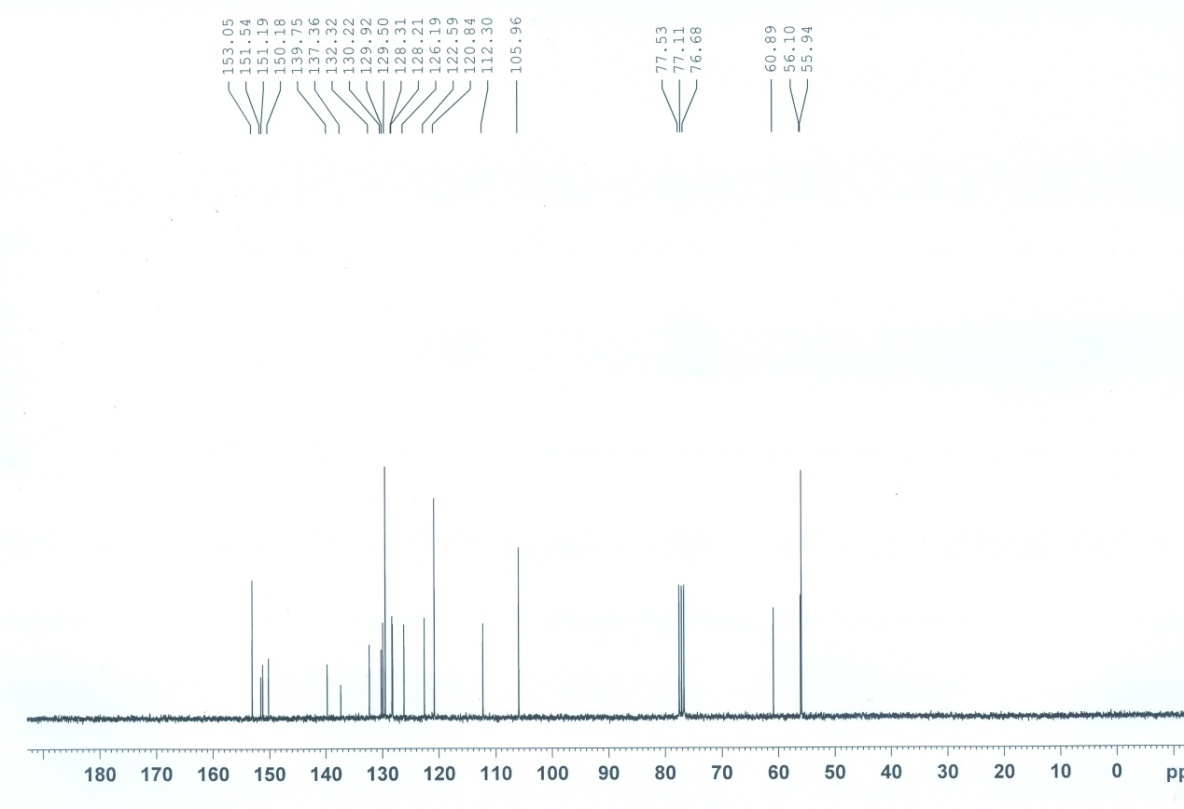


**^13^C NMR for compound 11**

***(Z)- 2-methoxy-5-(3, 4, 5-trimethoxystyryl)phenyl benzyl carbonic ester (12)***

Pale yellow syrup, yield:72%.^1^H-NMR (300 MHz, CDCl_3_) *δ*: 7.30-7.45 (m, 5H, H-2’’, H-3’’, H-4’’, H-5’’, H-6’’), 7.09-7.15 (m, 2H, H-2’, H-6’), 6.85 (d, 1H, *J* = 8.4Hz, H-5’), 6.49 (s, 2H, H-2, H-6), 6.45 (s, 2H, H-a, H-a’), 5.24 (s, 2H, 7’’-OCH_2_), 3.83 (s, 3H, OCH_3_), 3.78 (s, 3H, OCH_3_), 3.69 (s, 6H, OCH_3_×2); ^13^C-NMR (75 MHz, CDCl_3_) *δ*: 153.2, 153.0, 150.3, 139.9, 137.3, 134.9, 132.3, 130.1, 129.7, 128.6, 128.4, 128.3, 127.9, 122.7, 112.2, 105.9, 70.3, 60.9, 56.0, 55.9; HRMS (ESI) *m/z*: calcd for C_26_H_26_O_7_Na (M+Na) 473.1577, found473.1591 [M+Na]^+^.

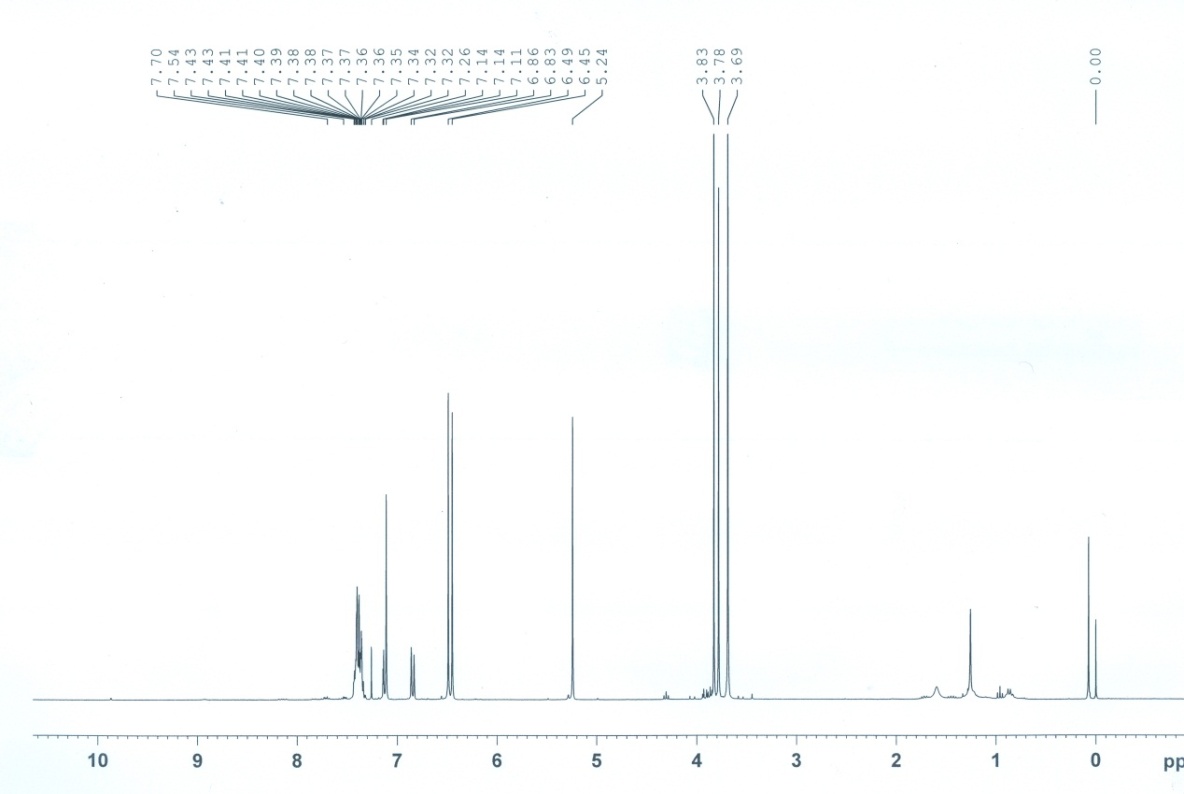


**^1^HNMR for compound 12**


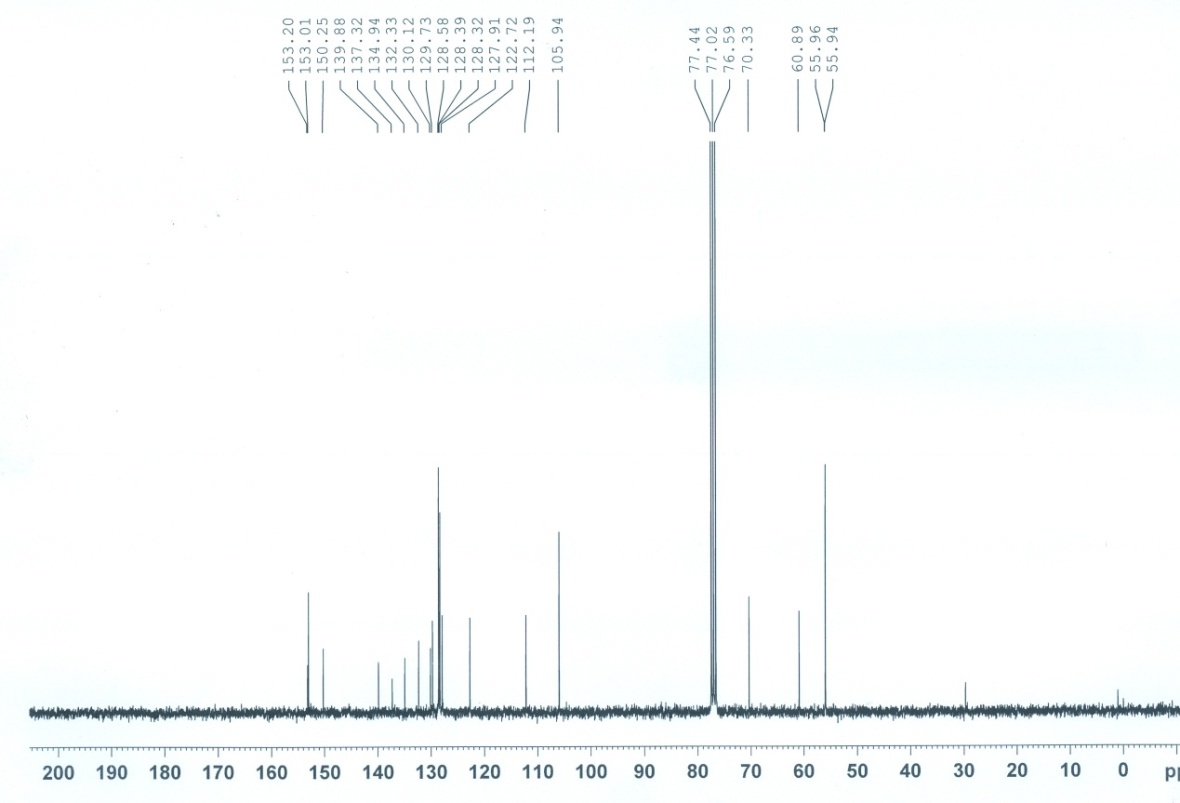


**^13^C NMR for compound 12**

***(Z)-2-methoxy-5-(3, 4, 5-trimethoxystyryl)phenyl dodecyl carbonic ester (13)***

Pale yellow syrup, yield:68%.^1^H-NMR (300 MHz, CDCl3) *δ*: 7.10-7.15 (m, 2H, H-2’, H-6’), 6.85 (d, 1H, *J* = 8.1Hz, H-5’), 6.49 (s, 2H, H-2, H-6), 6.46 (s, 2H, H-a, H-a’), 4.21 (t, 2H, *J* = 6.9Hz, OCH_2_), 3.84 (s, 3H, OCH_3_), 3.82 (s, 3H, OCH_3_), 3.70 (s, 6H, OCH_3_×2), 1.72 (m, 2H, CH_2_), 1.20-1.45 (m, 18H, CH_2_×9), 0.88 (t, 3H, *J* = 6.9Hz, CH_3_); ^13^C-NMR (75 MHz, CDCl_3_) *δ*: 153.3, 153.0, 150.3, 139.9, 137.3, 132.4, 130.1, 129.7, 128.4, 127.8, 122.8, 112.2, 105.9, 69.1, 60.9, 56.0, 55.9, 31.9, 29.6, 29.5, 29.4, 29.3, 29.2, 28.6, 25.6, 22.7, 14.1; HRMS (ESI) *m/z*: calcd for C_31_H_44_O_7_Na (M+Na)551.2986, found551.2997 [M+Na]^+^.

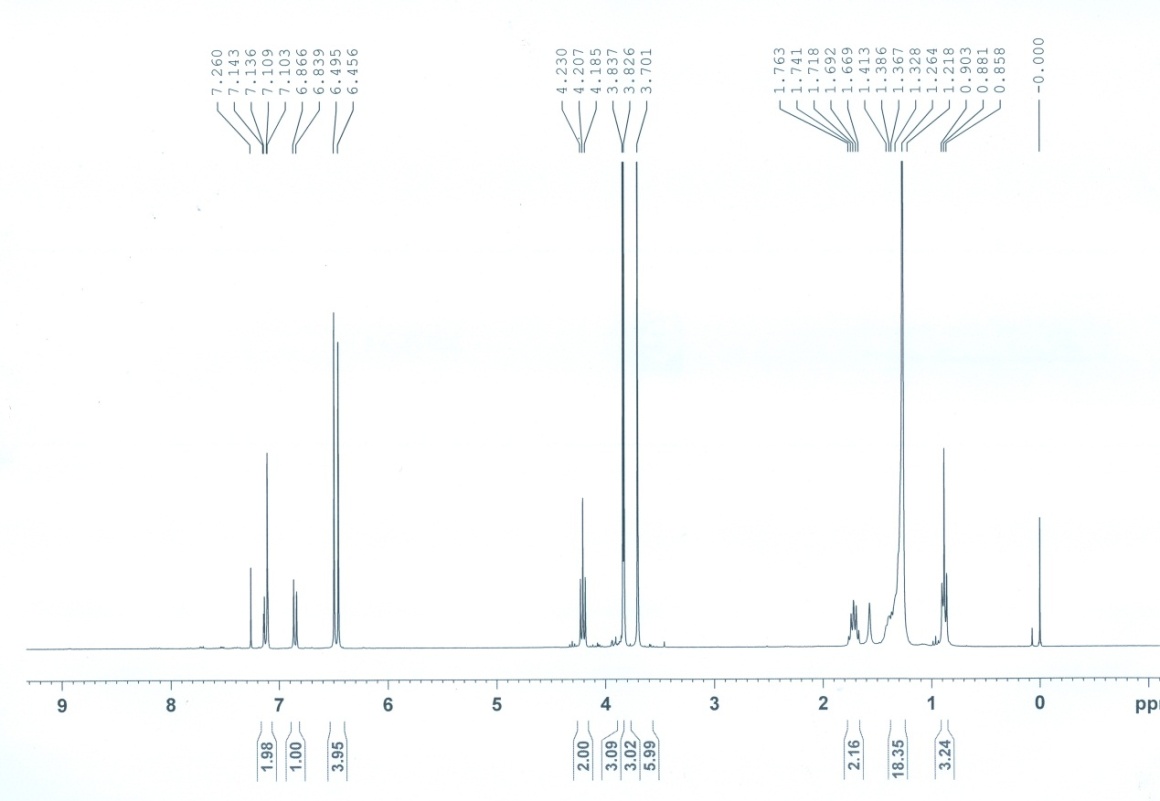


**^1^H NMR for compound 13**


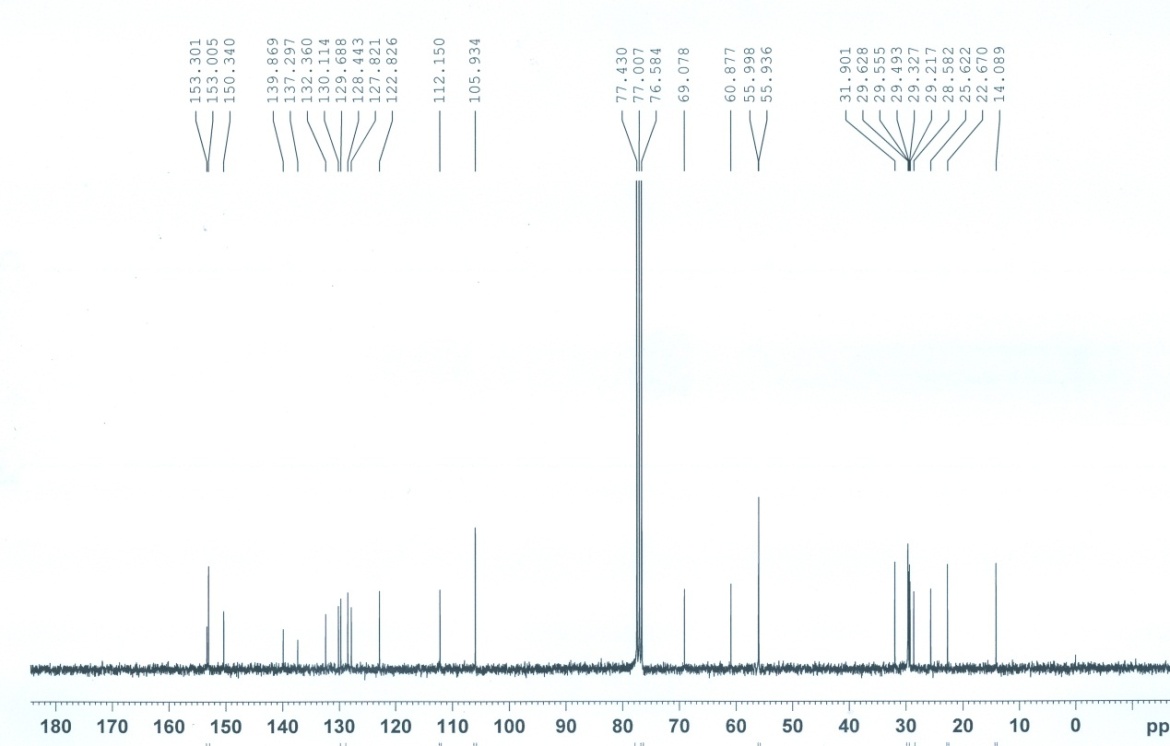


**^13^C NMR for compound 13**

***(Z)-2-methoxy-5-(3, 4, 5-trimethoxystyryl)phenyl hexadecyl carbonic ester (14)***

Pale yellow solid, m.p. 46-48°C, yield: 65%. ^1^H-NMR (300 MHz, CDCl_3_) *δ*: 6.95-7.15 (m, 2H, H-2’, H-6’), 6.85 (d, 1H, *J* = 8.4Hz, H-5’), 6.49(s, 2H, H-2, H-6), 6.45 (s, 2H, H-a, H-a’), 4.21 (t, 2H, *J* = 6.9Hz, OCH_2_), 3.85 (s, 3H, OCH_3_), 3.82 (s, 3H, OCH_3_), 3.70 (s, 6H, OCH_3_×2), 1.72 (m, 2H, CH_2_), 1.20-1.47 (m, 26H, CH_2_×13), 0.88 (t, 3H, *J* = 6.9Hz, CH_3_); ^13^C-NMR (75MHz, CDCl_3_) *δ*: 153.3, 153.0, 150.3, 139.9, 137.3, 132.4, 130.1, 129.7, 128.4, 127.8, 122.8, 112.1, 105.9, 69.1, 60.9, 56.0, 55.9, 31.9, 29.7, 29.6, 29.5, 29.4, 29.3, 29.3, 28.6, 25.6, 22.7, 14.1; HRMS (ESI) *m/z*: calcd for C_35_H_52_O_7_Na (M+Na) 607.3612, found 607.3626 [M+Na]^+^.

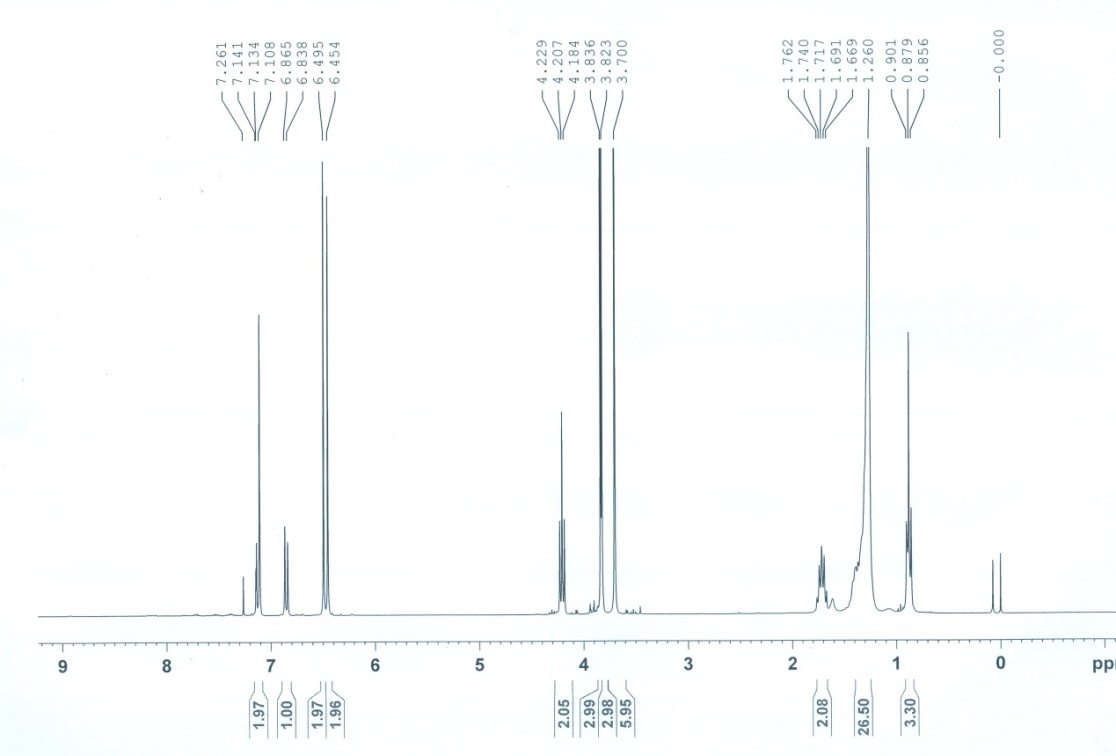


**^1^H NMR for compound 14**


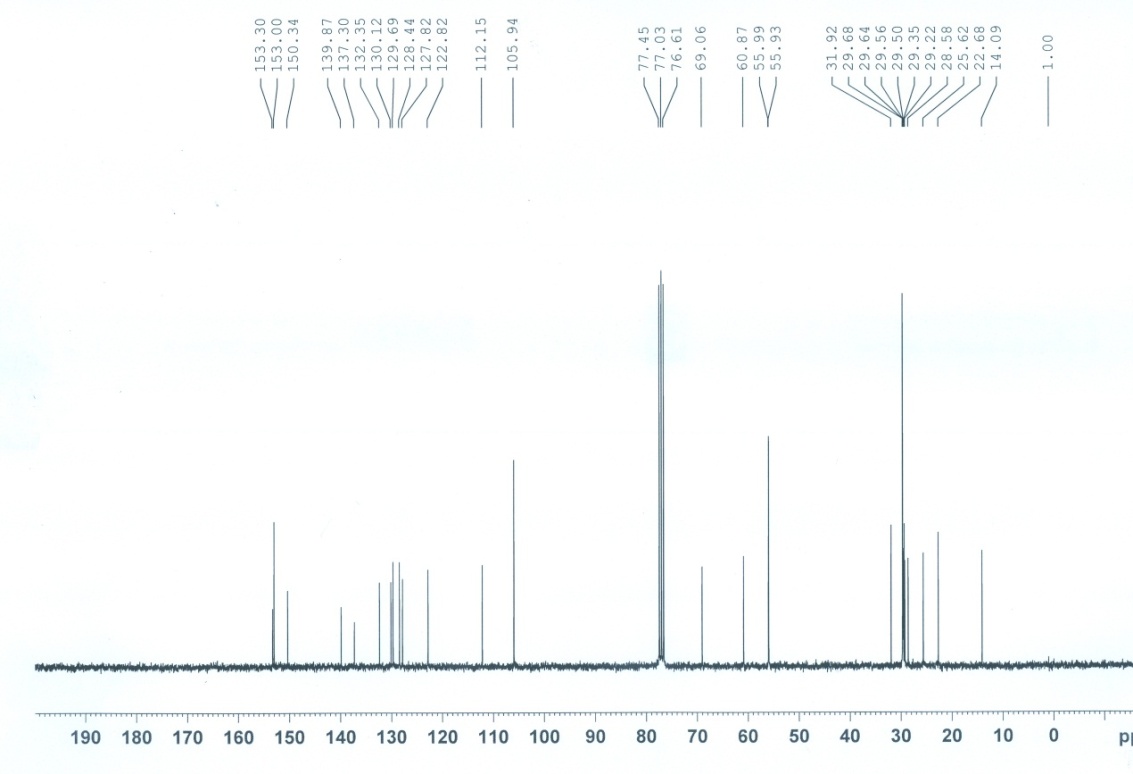


**^13^C NMR for compound 14**
